# The algebraic characterizations for a formal power series over complete strong bimonoids

**DOI:** 10.1186/s40064-016-1764-x

**Published:** 2016-03-10

**Authors:** Jian Hua Jin, Chun Quan Li

**Affiliations:** College of Sciences, Southwest Petroleum University, Xindu District, Xindu Road, Chengdu, 610500 China; College of Mathematics and Econometrics, Hunan University, Yuelu Mountain South Road, Changsha, 410082 China

**Keywords:** Formal power series, Equivalence, Weighted pushdown automata, Weighted context-free grammars, Strong bimonoids

## Abstract

On the basis of run semantics and breadth-first algebraic semantics, the algebraic characterizations for a classes of formal power series over complete strong bimonoids are investigated in this paper. As recognizers, weighted pushdown automata with final states (WPDAs for short) and empty stack (WPDAs$^{\emptyset }$) are shown to be equivalent based on run semantics. Moreover, it is demonstrated that for every WPDA there is an equivalent crisp-simple weighted pushdown automaton with final states by run semantics if the underlying complete strong bimonoid satisfies multiplicatively local finiteness condition. As another type of generators, weighted context-free grammars over complete strong bimonoids are introduced, which are proven to be equivalent to WPDAs$^{\emptyset }$ based on each one of both run semantics and breadth-first algebraic semantics. Finally examples are presented to illuminate the proposed methods and results.

## Background

Weighted automata (Droste et al. 2009) are classical automata in which the transitions carry weights. These weights can be modeled as the cost involved when executing the transition, the probability or reliability of its successful execution. The weight algebraic structures are often described as semirings, therefore weighted automata have a rich structure theory and also result in recent practical applications in digital image compression (Culik and Kari 1993), natural language processing (Knight and May 2009; Mohri 1997) and model checking (Albert and Kari 2009; Meinecke and Quaas 2014).

A semiring consists of a set with two operations addition and multiplication satisfying certain natural axioms like associativity, commutativity and distributivity, just like the natural numbers with their laws for sums and products. Bounded distributive lattices, semiring-reducts of lattice-ordered monoids and of complete residuated lattices, and Brouwerian lattices are semirings. A strong bimonoid is a more general structure that can be viewed as a semiring where the distributivity assumption is dropped. All semirings are also strong bimonoids, but there are many typical examples of strong bimonoids which are not semirings such as bounded lattices, orthomodular lattices as a basis of quantum logic, and the interval [0,1] with *t*-norm and *t*-conorm from multi-valued logic. Recently, it has aroused considerable interest to investigate weighted automata with truth values in these strong bimonoids. For instance, fuzzy automata and fuzzy tree automata, defined by a pair of a *t*-norm and a *t*-conorm on the real unit interval, were discussed by Bozapalidis and Louscou-Bozapalidou (2006, 2008, 2010). Automata based on quantum logics were investigated in detail by researchers respectively (Qiu 2004, 2007a, b; Qiu and Ying 2004; Ying 2000a, b, 2005). Finite automata theory with membership values in lattices were established by Li (2011), where the role of the distributive law for the underlying lattice was analyzed. Droste et al. (2010) established weighted finite automata theory over strong bimonoids based on run semantics, initial algebra semantics and the free monoid semantics, which generalized several results from the references (Ignjatović et al. 2008; Li and Pedrycz 2005) derived for automata over lattice-ordered monoids or semiring-reducts of residuated lattices. Ćirić et al. (2010) presented determinization of weighted finite automata over strong bimonoids based on three different semantics including run semantics, initial algebra semantics and transition semantics.

It is well known that pushdown automata are another kind of important computational models and have more power than classical finite automata (Hopcroft and Ullman 1979). Xing (2007) studied fuzzy pushdown automata and fuzzy context-free languages based on lattice-ordered structures. Xing et al. (2009) introduced pushdown automata theory based on complete residuated lattice-valued logic and showed that the class of the languages accepted by pushdown automata with empty stack coincides with that accepted by pushdown automata with final states over complete residuated lattice-valued logic. Then pumping lemma in context-free grammar theory based on complete residuated lattice-valued logic was also established (Xing and Qiu 2009). It is the goal of this paper to investigate the algebraic characterizations for a power formal series over complete strong bimonoids, which could be generated by some weighted pushdown automaton or some weighted context-free grammars over complete strong bimonoids. Furthermore, we want to know how about these machines’ behaviors based on the proposed run semantics and breadth-first algebraic semantics. It may be very useful to know whether much more general results than weighted automata over strong bimonoids are obtained.

The remaining part of the paper is organized as follows. Based on run semantics and breadth-first algebraic semantics, we investigate weighted pushdown automata over complete strong bimonoids with final states and empty stack respectively and their recognizable languages. Sufficient conditions are proposed under which these machines’ behaviors coincide. Taking complete strong bimonoids as the structures of truth values, the notion of weighted context-free grammars (WCFGs) is then introduced. It is demonstrated that, based on each one of both run semantics and breadth-first algebraic semantics, WPDAs$^{\emptyset }$ and WCFGs are equivalent in the sense that they generate the same classes of formal power series. Examples are also given to illustrate the proposed methods and results. Finally conclusions and future work are presented.

## Weighted pushdown automata over complete strong bimonoids

A bimonoid is a structure $(A, +, \cdot , 0, 1)$ consisting of a set *A*, two binary operations $+$ and $\cdot$ on *A* and two constants $0,\ 1\in A$ such that $(A,+,0)$ and $(A,\cdot ,1)$ are monoids associated with the identity elements 0 and 1 respectively. Moreover, a bimonoid $(A, +, \cdot , 0, 1)$ is called a strong bimonoid if $+$ is commutative and $a\cdot 0=0\cdot a=0$ for every $a\in A$. Next the strong bimonoid $(A, +, \cdot , 0, 1)$ is usually abbreviated as *A* if no confusion arises. A strong bimonoid *A* is said to be right distributive if it satisfies $(a+b)\cdot c=a\cdot c+b\cdot c$ for every $a,b,c\in A$. A strong bimonoid *A* is said to be left distributive if it satisfies $c\cdot (a+b)=c\cdot a+c\cdot b$ for every $a,b,c\in A$. Then a semiring is a strong bimonoid which is left and right distributive.

A monoid $(A,+,0)$ is complete if it has an infinite sum operation $\sum _{I}{:}\; A^{I}\rightarrow A$ for any index set *A* such that $\sum _{i\in \emptyset }a_{i}=0$, $\sum _{i\in \{k\}}a_{i}=a_{k}$, $\sum _{i\in \{j,k\}}a_{i}=a_{j}+a_{k}$ for $j\ne k$, and $\sum _{j\in J}\left( \sum _{i\in I_{j}}a_{i}\right) =\sum _{i\in I}a_{i}$ if $\bigcup _{j\in J}I_{j}=I$ and $I_{j}\cap I_{k}=\emptyset$ for $j\ne k$. A monoid $(A,+,0)$ is idempotent if $a+a=a$ for any $a\in A$. A complete monoid $(A,+,0)$ is completely idempotent if $\sum _{I}a=a$ for any $a\in A$ and any index set *I*. If a strong monoid $(A,+,\cdot ,0,1)$ is complete, idempotent or completely idempotent, then $(A,+,\cdot ,0,1)$ is called a complete strong bimonoid, an idempotent strong bimonoid or a completely idempotent strong bimonoid respectively.

Let $\Sigma ^{*}$ be a free monoid generated from a finite nonempty set $\Sigma$ with the operator of concatenation, where the empty string $\varepsilon$ is identified with the identity of $\Sigma ^{*}$. A formal power series is a mapping $f:\Sigma ^{*}\rightarrow A$. *Im*(*f*) denotes the image set of *f*, i.e., $Im(f)=\{f(\omega )|\omega \in \Sigma ^{*}\}$. $|\Sigma |$ denotes the cardinality of a set $\Sigma$.

Next we introduce weighted pushdown automata over strong bimonoids on the basis of run semantics and breadth-first algebraic semantics. Then we investigate conditions under which these behaviors coincide.

### **Definition 1**

A weighted pushdown automaton with final states (WPDA for short) over a complete strong bimonoid *A* is a seven tuple $\mathcal {M}=(Q,\Sigma ,\Gamma ,\delta ,I,Z_{0},F)$, where

(i) *Q* is a finite nonempty set of states;
(ii) $\Sigma$ is a finite nonempty set of input symbols;
(iii) $\Gamma$ is a finite nonempty set of stack symbols;
(iv) $$\delta $$ is a mapping from $$Q\times (\Sigma \cup \{\varepsilon \})\times \Gamma \times Q\times \Gamma ^{*}$$ to *A* and the set $$\{(q,\tau ,Z,p,\gamma )|\delta (q,\tau ,Z,$$$$p,\gamma )\in A{\setminus }\{0\},\ (q,\tau ,Z,p,\gamma )\in Q\times (\Sigma \cup \{\varepsilon \})\times \Gamma \times Q\times \Gamma ^{*}\}$$ is finite, where $$\delta (q,\tau ,Z,p,\gamma )$$ expresses the truth value of the transition that inputting $$\tau $$ makes state *q* transfer to state *p*, replace the stop symbol *Z* on the stack by string $$\gamma $$ and advance the input head one symbol;
(v) $Z_{0}\in \Gamma$ is called the start stack symbol;
(vi) *I* and *F* are mappings from *Q* to *A*, which are called the weighted subsets of initial and final states respectively.

### **Definition 2**

A weighted pushdown automaton with empty stack (WPDA$^{\emptyset }$ for short) over a complete strong bimonoid *A* is a seven tuple $\mathcal {N}=(Q,\Sigma ,\Gamma ,\delta ,I,Z_{0},\emptyset )$, where *Q*, $\Sigma$, $\Gamma$, $\delta$, *I* and $Z_{0}$ are the same as those in WPDA $\mathcal {M}$, and $\emptyset$ represents an empty set.

To describe the behavior of a weighted pushdown automaton, it is necessary to introduce the concept of an instantaneous description. An instantaneous description is a three-tuple $(q,\omega ,\gamma )\in Q\times \Sigma ^{*}\times \Gamma ^{*}$, which means that the automaton is in the state *q* and has unexpended input $\omega$ and stack contents $\gamma$. An instantaneous description represents the configuration of a WPDA at a given instant. Let $D=Q\times \Sigma ^{*}\times \Gamma ^{*}$. To introduce the transition in a WPDA in terms of instantaneous descriptions, we define $\vdash _{\mathcal {M}}$ as a mapping from $D\times D$ to *A*.

### **Definition 3**

Let $\mathcal {M}=(Q,\Sigma ,\Gamma ,\delta ,I,Z_{0},F)$ be a WPDA over a complete strong bimonoid *A*. Define $\vdash _{\mathcal {M}}$ as a mapping from $D\times D$ to *A* in the form of:

$$\begin{aligned} \vdash _{\mathcal {M}}((q,\omega ,\beta ),(p,u,\alpha )) =\left\{ \begin{array}{ll} b, &{}\quad \hbox {if }u=\omega , \, tail(\beta )\le \alpha \\ c, &{}\quad \hbox { if }u=tail(\omega ),\, tail(\beta )\le \alpha \\ 0, &{}\quad \hbox { otherwise} \end{array}\right. \end{aligned}$$

for any $(q,\omega ,\beta ),(p,u,\alpha )\in D$, where

$$\begin{aligned} b=\delta (q,\varepsilon ,head(\beta ),p,\alpha {\setminus } tail(\beta )) \end{aligned}$$

and

$$\begin{aligned} c=\delta (q,head(\omega ),head(\beta ),p,\alpha {\setminus } tail(\beta )). \end{aligned}$$

For every nonempty string $u=xu_{1}\in \Sigma ^{*}$, $x\in \Sigma$, head$(u)=x$ and tail$(u)=u_{1}$. If there exists $\gamma \in \Gamma ^{*}$ such that $\alpha =\gamma \beta$, then $\beta \le \alpha$ and we denote $\gamma =\alpha {\setminus }\beta$.

For a WPDA $\mathcal {M}=(Q,\Sigma ,\Gamma ,\delta ,I,Z_{0},F)$ over a complete strong bimonoid *A*, we define two different semantics, called run semantics and breadth-first algebraic semantics. For the run semantics, the weight of a string $\omega$ is computed by summing up the weights of all successful runs of $\mathcal {M}$ on $\omega$ where the weight of a run is the product of the weights of the involved transitions.

Formally, the *r*-behavior of $$\mathcal {M}$$, denoted by $$\langle \mathcal {M}\rangle _{r}$$, is a mapping from $$\Sigma ^{*}$$ to *A*, defined for every $$\omega \in \Sigma ^{*}$$ by letting $$\langle \mathcal {M}\rangle _{r}(\omega )=\sum \{I(q_{0})\cdot \vdash _{\mathcal {M}}((q_{0},\omega ,Z_{0}),(q_{1},\sigma _{2}\ldots \sigma _{n},$$$$Z_{1}\gamma _{1})) \cdot \vdash _{\mathcal {M}}((q_{1},\sigma _{2}\ldots \sigma _{n},$$$$Z_{1}\gamma _{1}), (q_{2},\sigma _{3}\ldots \sigma _{n},Z_{2}\gamma _{2}))\ldots \vdash _{\mathcal {M}}((q_{n-1},\sigma _{n},Z_{n-1}\gamma _{n-1}), (q_{n},\varepsilon ,Z_{n}\gamma _{n}))\cdot F(q_{n})|\omega =\sigma _{1}\ldots \sigma _{n},$$$$\sigma _{i}\in \Sigma \cup \{\varepsilon \}$$, $$i=1,\ldots ,n$$, $$(q_{0},q_{1},\ldots ,q_{n})\in Q^{n+1}$$, $$Z_{1},\ldots ,Z_{n-1}\in \Gamma $$, $$\gamma _{1},\ldots ,\gamma _{n}\in \Gamma ^{*}$$, $$Z_{n}\in \Gamma ^{*}\}$$.

To determine the breadth-first algebra behavior of $\mathcal {M}$ for $\omega \in \Sigma ^{*}$, we start with the initial weighted states *I*, execute $\omega$, and apply the final weighted states *F* at the end.

The *b*-behavior of $$\mathcal {M}$$, denoted by $$\langle \mathcal {M}\rangle _{b}$$, is a mapping from $$\Sigma ^{*}$$ to *A*, defined for every $$\omega \in \Sigma ^{*}$$ by letting $$\langle \mathcal {M}\rangle _{b}(\omega )=\sum \{I(q_{0})\cdot (\sum \{\vdash _{\mathcal {M}}((q_{0},\omega ,Z_{0}),(q_{1},\sigma _{2}\ldots $$$$\sigma _{n}, Z_{1}\gamma _{1})) \cdot \vdash _{\mathcal {M}}((q_{1},\sigma _{2}\ldots \sigma _{n},$$$$Z_{1}\gamma _{1}), (q_{2},\sigma _{3}\ldots \sigma _{n},Z_{2}\gamma _{2})) \ldots \vdash _{\mathcal {M}}((q_{n-1},\sigma _{n},Z_{n-1}\gamma _{n-1}), (q_{n},\varepsilon ,Z_{n}\gamma _{n}))|\omega =\sigma _{1}\ldots \sigma _{n},$$$$\sigma _{i}\in \Sigma \cup \{\varepsilon \}$$, $$i=1,\ldots ,n$$, $$(q_{1},\ldots ,q_{n-1})\in Q^{n-1}$$, $$Z_{1},\ldots ,Z_{n-1}\in \Gamma $$, $$\gamma _{1},\ldots ,\gamma _{n}\in \Gamma ^{*}$$, $$Z_{n}\in \Gamma ^{*}\})\cdot F(q_{n})|$$$$q_{0},q_{n}\in Q\}$$.

For a WPDA$$^{\emptyset }$$$$\mathcal {N}=(Q,\Sigma ,\Gamma ,\delta ,I,Z_{0},\emptyset )$$ over *A*, the *r*-behavior of $$\mathcal {N}$$, denoted by $$\langle \mathrm{rec}$$$$(\mathcal {N})\rangle _{r}$$, is a mapping from $$\Sigma ^{*}$$ to *A*, defined for every $$\omega \in \Sigma ^{*}$$ by letting $$\langle \mathrm{rec}$$$$(\mathcal {N})\rangle _{r}(\omega )=\sum \{I(q_{0})\cdot \vdash _{\mathcal {N}}((q_{0},\omega ,Z_{0}),(q_{1},\sigma _{2}\ldots \sigma _{n},$$$$ Z_{1}\gamma _{1}))\cdot \vdash _{\mathcal {N}}((q_{1},\sigma _{2}\ldots \sigma _{n},$$$$Z_{1}\gamma _{1}), (q_{2},\sigma _{3}\ldots \sigma _{n},Z_{2}\gamma _{2}))\ldots \vdash _{\mathcal {N}}((q_{n-1},\sigma _{n},Z_{n-1}\gamma _{n-1}), (q_{n},\varepsilon ,\varepsilon ))|\omega =\sigma _{1}\ldots \sigma _{n},$$$$\sigma _{i}\in \Sigma \cup \{\varepsilon \}$$, $$i=1,\ldots ,n$$, $$(q_{0},q_{1},\ldots ,q_{n})\in Q^{n+1}$$, $$Z_{1},\ldots ,Z_{n-1}\in \Gamma $$, $$\gamma _{1},\ldots ,\gamma _{n-1}\in \Gamma ^{*}\}$$.

The breadth-first algebraic behavior of $$\mathcal {N}$$, denoted by $$\langle \mathrm{rec}$$$$(\mathcal {N})\rangle _{b}$$, is a mapping from $$\Sigma ^{*}$$ to *A*, defined for every $$\omega \in \Sigma ^{*}$$ by $$\langle \mathrm{rec}$$$$(\mathcal {N})\rangle _{b}(\omega )=\sum \{I(q_{0})\cdot (\sum \{\vdash _{\mathcal {N}}((q_{0},\omega ,Z_{0}),(q_{1},\sigma _{2}$$$$\ldots \sigma _{n}, Z_{1}\gamma _{1})) \cdot \vdash _{\mathcal {N}}((q_{1},\sigma _{2}\ldots $$$$\sigma _{n},Z_{1}\gamma _{1}), (q_{2},\sigma _{3}\ldots \sigma _{n},Z_{2}\gamma _{2}))$$$$\ldots \vdash _{\mathcal {N}}((q_{n-1},\sigma _{n},Z_{n-1}\gamma _{n-1}), (q_{n},\varepsilon ,\varepsilon ))|\omega =\sigma _{1}\ldots \sigma _{n},$$$$\sigma _{i}\in \Sigma \cup \{\varepsilon \}$$, $$i=1,\ldots ,n$$, $$(q_{1},\ldots ,q_{n})\in Q^{n}$$, $$Z_{1},\ldots ,$$$$Z_{n-1}\in \Gamma $$, $$\gamma _{1},\ldots ,\gamma _{n-1}\in \Gamma ^{*}$$$$\})|q_{0}\in Q\}$$.

Let $$x\in \{r,b\}$$. Then a formal power series $$f:\Sigma ^{*}\rightarrow A$$ is *x*-recognizable if there is a WPDA $$\mathcal {M}$$ or WPDA$$^{\emptyset }$$$$\mathcal {N}$$ over *A* such that $$\langle \mathcal {M}\rangle _{x}=f$$ or $$\langle \mathrm{rec}$$$$(\mathcal {N})\rangle _{x}=f$$. We say that two WPDAs $$\mathcal {M}$$ and $$\mathcal {M}^{'}$$ over *A* are *x*-equivalent if $$\langle \mathcal {M}\rangle _{x}=\langle \mathcal {M}^{'}\rangle _{x}$$.

A WPDA over *A* is essentially a weighted finite automaton over *A* with $\varepsilon$-transition and an addition of a stack, on which it can store a string of stack symbols. Droste et al. (2010) introduced weighted finite automata (WFAs) over *A* and investigated their behaviors on the basis of run semantics and initial algebra semantics. Let $\mathcal {M}_{1}=(Q,\Sigma ,\delta ,I,F)$ be a WFA over *A*, where *Q* and $\Sigma$ are the sets of finite nonempty states and input symbols respectively; *I* and *F* are the mappings from *Q* to *A*, called the weighted subsets of initial and final states respectively; and $\delta$ is a mapping from $Q\times \Sigma \times Q$ to *A*.

In order to compute with words, the extension of $\delta$, denoted by the mapping $\delta ^{*}{:}\;Q\times \Sigma ^{*}\times Q\rightarrow A$, is defined as follows: for any $q_{0},q_{n}\in Q$, if $q_{0}=q_{n}$, then $\delta ^{*}(q_{0},\varepsilon ,q_{n})=1$; otherwise, $\delta ^{*}(q_{0},\varepsilon ,q_{n})=0$; and for any $\theta =a_{1}\ldots a_{n}\in \Sigma ^{*}$ with $n\ge 1$, $\delta ^{*}(q_{0},\theta ,q_{n})=\sum \{\delta (q_{0},a_{1},q_{1})\ldots \delta (q_{n-1},a_{n},q_{n})|q_{1},\ldots ,q_{n-1}\in Q\}.$

For the run semantics, the language recognized by WFA $$\mathcal {M}_{1}$$ is a formal power series $$\mathcal {L}_{\mathcal {M}_{1}}^{r}:\Sigma ^{*}\rightarrow A$$, given by for every $$ \theta =a_{1}\ldots a_{n}\in \Sigma ^{*}{\setminus } \{\varepsilon \},$$$$a_{i}\in \Sigma {\setminus }\{\varepsilon \},$$$$i=1,\ldots ,n$$, $$\mathcal {L}_{\mathcal {M}_{1}}^{r}(\varepsilon )=\sum \{I(q)\cdot \delta ^{*}(q,\varepsilon ,q)\cdot F(q)|q\in Q\}$$ and $$\mathcal {L}_{\mathcal {M}_{1}}^{r}(\theta )=\sum \{I(q_{0})\cdot \delta (q_{0},a_{1},q_{1})\ldots \delta (q_{n-1},a_{n},q_{n})\cdot F(q_{n})|q_{0},\ldots ,q_{n}\in Q\}.$$

For the breadth-first algebraic semantics, the language recognized by WFA $\mathcal {M}_{1}$ is a formal power series $\mathcal {L}_{\mathcal {M}_{1}}^{b}:\Sigma ^{*}\rightarrow A$, given by for every $\theta \in \Sigma ^{*}$, $\mathcal {L}_{\mathcal {M}_{1}}^{b}(\theta )=\sum \{I(q)\cdot \delta ^{*}(q,\theta ,p)\cdot F(p)|q,p\in Q\}.$

Noting that a bounded lattice $L=(L,\vee ,\wedge ,0,1)$ is a strong bimonoid. Jin et al. (2012) have presented that the run semantics and the breadth-first algebraic semantics differ for a given WFA over a strong bimonoid *L*. Moreover, when compared with WPDAs over a general strong bimonoid *A*, the behaviors of WFAs over *A* are shown no more power regardless of run semantics or the breadth-first algebraic semantics as follows.

### **Proposition 1**

*Let a formal power series*$f_{x}{:}\;\Sigma ^{*}\rightarrow A$*bex*-*recognizable by a weighted finite automaton over a complete strong bimonoidA*, *where*$x\in \{r,b\}$. *Then*$f_{x}$*is alsox*-*recognizable by a certain WPDA*$\mathcal {M}$*overA*.

### *Proof*

Assume that $f_{x}$ is *x*-recognizable by a WFA $\mathcal {M}_{1}=(Q,\Sigma ,\delta ,I,F)$ over *A*. Construct a WPDA $\mathcal {M}=(Q,\Sigma ,\{Z\},\eta ,I,Z,F)$ as follows:

For every $q,q^{'}\in Q$ and $\tau \in \Sigma$, $\eta (q,\tau ,Z,q^{'},Z)=\delta (q,\tau ,q^{'})$, $\eta (q,\varepsilon ,Z,q,Z)=1;$ otherwise, $\eta (q,a,Z,q^{'},\gamma )=0$ for $a\in \Sigma \cup \{\varepsilon \}$ and $\gamma \in \{Z\}^{*}{\setminus } \{Z\}$.

Then for every $$\omega =\sigma _{1}\ldots \sigma _{n}\in \Sigma ^{*}{\setminus }\{\varepsilon \}$$, $$\sigma _{i}\in \Sigma $$, $$i=1,\ldots ,n,$$ we have $$f_{r}(\omega )=\sum \{I(q_{0})\cdot \delta (q_{0},\sigma _{1},q_{1})\ldots \delta (q_{n-1},\sigma _{n},q_{n})\cdot F(q_{n})|q_{0},\ldots ,q_{n}\in Q\}$$$$=\sum \{I(q_{0})\cdot \vdash _{\mathcal {M}}((q_{0},\omega ,Z),(q_{1},$$$$\sigma _{2}\ldots \sigma _{n},$$$$Z)) \ldots \vdash _{\mathcal {M}}((q_{n-1},\sigma _{n},Z), (q_{n},\varepsilon ,Z))\cdot F(q_{n})|$$$$(q_{0},q_{1},\ldots ,q_{n})\in Q^{n+1}\}=\langle \mathcal {M}\rangle _{r}(\omega )$$, $$f_{b}(\omega )=\sum \{I(q_{0})\cdot \delta ^{*}(q_{0},\omega ,q_{n})\cdot F(q_{n})|q_{0},q_{n}\in Q\}$$$$=\sum \{I(q_{0})\cdot (\sum \{\delta (q_{0},\sigma _{1},q_{1}) \ldots \delta (q_{n-1},\sigma _{n},q_{n})|q_{1},\ldots ,q_{n-1}$$$$\in Q\})\cdot F(q_{n})|q_{0},q_{n}\in Q\}$$$$=\sum \{I(q_{0})\cdot (\sum \{\vdash _{\mathcal {M}}((q_{0},\omega ,Z),$$$$(q_{1},\sigma _{2}\ldots \sigma _{n},$$$$Z)) \ldots \vdash _{\mathcal {M}}((q_{n-1},\sigma _{n},Z), (q_{n},\varepsilon ,Z))|q_{1},$$$$\ldots ,q_{n-1}\in Q\})\cdot F(q_{n})|$$$$(q_{0},q_{1},\ldots ,q_{n})\in Q^{n+1}\}=\langle \mathcal {M}\rangle _{r}(\omega )$$, and $$f_{r}(\varepsilon )=f_{b}(\varepsilon )=\sum \{I(q)\cdot \delta ^{*}(q,\varepsilon ,q)\cdot F(q)|q\in Q\}=\sum \{I(q)\cdot \vdash _{\mathcal {M}}((q,\varepsilon ,Z), (q,\varepsilon ,Z))\cdot F(q)|q\in Q\}=\langle \mathcal {M}\rangle _{b}(\varepsilon )$$$$=\langle \mathcal {M}\rangle _{r}(\varepsilon )$$.

Hence $f_{x}$ is also *x*-recognizable by a certain WPDA $\mathcal {M}$ over *A*.

By a certain semantic way, two weighted pushdown automata are considered equivalent if they can recognize the same classes of fomal power series. Next we will prove that WPDAs and WPDAs$^{\emptyset }$ over a complete strong bimonoid *A* are equivalent based on the run semantics. $\square$

### **Proposition 2**

*Letfbe a formal power series from*$\Sigma ^{*}$*to a complete strong bimonoidA*. *Then the following statements are equivalent:*

1. *fisr*-*recognizable by a certain WPDA overA*;
2. *fisr*-*recognizable by a certain*$\hbox {WPDA}^{\emptyset }$*overA*.

### *Proof*

(1) implies (2): Let *f* be *r*-recognizable by a WPDA $$\mathcal {M}=(Q, \Sigma ,\Gamma ,\delta ,I,Z_{0},F)$$ over *A*. Now construct a WPDA$$^{\emptyset }$$$$\mathcal {N}=(Q^{'},\Sigma , \Gamma ^{'},\delta ^{'},I^{'},X_{0},\emptyset )$$ such that $$\langle \mathrm rec$$$$(\mathcal {N})\rangle _{r}=\langle \mathcal {M}\rangle _{r}$$, where $$Q^{'}=Q\cup \{q_{0},q_{e}\}$$, $$q_{0},q_{e}\notin Q$$, $$\Gamma ^{'}=\Gamma \cup \{X_{0}\}$$, $$X_{0}\notin \Gamma $$, $$I^{'}:Q^{'}\rightarrow A$$ is a mapping defined by letting $$I^{'}(q_{0})=1$$, $$I^{'}(q_{e})=0$$ and $$I^{'}(q)=0$$ for $$q\in Q$$, and $$\delta ^{'}:Q^{'}\times (\Sigma \cup \{\varepsilon \})\times \Gamma ^{'}\times Q^{'}\times \Gamma ^{'*}\rightarrow A$$ is a mapping defined by letting

(i) $$\delta ^{'}(q_{0},\varepsilon ,X_{0},q,Z_{0}X_{0})=I(q)$$, $$\delta ^{'}(q,\varepsilon ,Z,q_{e},\varepsilon )=F(q)$$, $$\delta ^{'}(q_{e},\varepsilon ,Z,q_{e},\varepsilon )=1$$, $$\forall q\in Q, Z\in \Gamma ^{'}$$;
(ii) $$\delta ^{'}(q,\tau ,X,p,\gamma )=\delta (q,\tau ,X,p,\gamma )$$, $$\forall q,p\in Q$$, $$\tau \in \Sigma \cup \{\varepsilon \}$$, $$X\in \Gamma $$, $$\gamma \in \Gamma ^{*}$$;
(iii) Otherwise, $\delta ^{'}(q,\tau ,X,p,\gamma )=0$ for $(q,\tau ,X,p,\gamma )\in Q^{'}\times (\Sigma \cup \{\varepsilon \})\times \Gamma ^{'}\times Q^{'}\times \Gamma ^{'*}$.

Next we prove $$\langle \mathrm{rec}$$$$(\mathcal {N})\rangle _{r}=\langle \mathcal {M}\rangle _{r}$$. In fact, for every $$\omega \in \Sigma ^{*}$$, we have $$\langle \mathrm{rec}$$$$(\mathcal {N})\rangle _{r}(\omega )=\sum \{I^{'}(q_{0})\cdot \vdash _{\mathcal {N}}((q_{0},\omega ,X_{0}),(q_{1},\sigma _{2}\ldots $$$$\sigma _{n},$$$$ Z_{1}\gamma _{1}))\cdot \vdash _{\mathcal {N}}((q_{1},\sigma _{2}\ldots \sigma _{n},$$$$Z_{1}\gamma _{1}), (q_{2},\sigma _{3}\ldots \sigma _{n},Z_{2}\gamma _{2}))\ldots \vdash _{\mathcal {N}}((q_{n-1},\sigma _{n},Z_{n-1}\gamma _{n-1}), (q_{n},\varepsilon ,\varepsilon ))|\omega =\sigma _{1}\ldots \sigma _{n},$$$$\sigma _{i}\in \Sigma \cup \{\varepsilon \}$$, $$i=1,\ldots ,n$$, $$(q_{0},q_{1},\ldots ,q_{n})\in Q^{'n+1}$$, $$Z_{1},\ldots ,Z_{n-1}\in \Gamma ^{'}$$, $$\gamma _{1},\ldots ,\gamma _{n-1}\in \Gamma ^{'*}\}$$$$=\sum \{\vdash _{\mathcal {N}}((q_{0},\omega ,$$$$X_{0}),(q,\omega ,Z_{0}X_{0}))\cdot \vdash _{\mathcal {N}}((q,\omega ,Z_{0}X_{0}), (q_{1},\sigma _{2}\ldots \sigma _{n}, Z_{1}\gamma _{1}X_{0}))$$$$\cdot \vdash _{\mathcal {N}}((q_{1},\sigma _{2}\ldots \sigma _{n},Z_{1}\gamma _{1}X_{0}), (q_{2},\sigma _{3}\ldots \sigma _{n},Z_{2}\gamma _{2}X_{0}))\ldots \vdash _{\mathcal {N}}((q_{n-1},\sigma _{n},Z_{n-1}\gamma _{n-1}X_{0}),$$$$(q_{n},\varepsilon ,\gamma _{n-1}X_{0}))\cdot \vdash _{\mathcal {N}}((q_{n},\varepsilon ,\gamma _{n-1}X_{0}), (q_{e},\varepsilon ,\alpha _{2}\ldots \alpha _{k}X_{0}))\cdot \vdash _{\mathcal {N}}((q_{e},\varepsilon ,\alpha _{2}\ldots $$$$\alpha _{k}X_{0}),$$$$ (q_{e},\varepsilon ,\alpha _{3}\ldots \alpha _{k}X_{0}))\ldots \vdash _{\mathcal {N}}((q_{e},\varepsilon ,X_{0}), (q_{e},\varepsilon ,\varepsilon ))$$$$| \omega =\sigma _{1}\ldots \sigma _{n},\sigma _{i}\in \Sigma \cup \{\varepsilon \}, i=1,\ldots ,n, (q,q_{1},\ldots ,q_{n})\!\in \! Q^{n+1}, Z_{1},\ldots ,Z_{n-1}\!\in \!\Gamma , \gamma _{1},\ldots ,\gamma _{n-2}\!\in \!\Gamma ^{*},\gamma _{n-1}=\alpha _{1}$$$$ \ldots \alpha _{k},\alpha _{j}\!\in \!\Gamma , j=1,\ldots ,k\}$$$$+$$$$\sum \{\vdash _{\mathcal {N}}((q_{0},\omega ,$$$$X_{0}),(q,$$$$\omega ,Z_{0}X_{0}))\cdot \vdash _{\mathcal {N}}((q,\omega ,Z_{0}X_{0}),(q_{1},\sigma _{2}\ldots \sigma _{n}, Z_{1}\gamma _{1}X_{0}))$$$$\cdot \vdash _{\mathcal {N}}((q_{1},\sigma _{2}\ldots \sigma _{n},Z_{1}\gamma _{1}X_{0}), (q_{2},\sigma _{3}\ldots \sigma _{n},$$$$Z_{2}\gamma _{2}X_{0}))\ldots \vdash _{\mathcal {N}}((q_{n-1},\sigma _{n},Z_{n-1}\gamma _{n-1}X_{0}), (q_{n},\varepsilon ,X_{0}))\cdot \vdash _{\mathcal {N}}((q_{n},\varepsilon ,X_{0}),$$$$(q_{e},\varepsilon ,\varepsilon ))| \omega =\sigma _{1}\ldots \sigma _{n},\sigma _{i}\in \Sigma \cup \{\varepsilon \}, i=1,\ldots ,n, (q,q_{1},$$$$\ldots ,q_{n})\in Q^{n+1}, Z_{1},\ldots ,Z_{n-1}\in \Gamma , \gamma _{1},\ldots , \gamma _{n-2}\in \Gamma ^{*},$$$$\gamma _{n-1}=\varepsilon \}=\sum \{I(q)\cdot \vdash _{\mathcal {M}}((q,\omega ,Z_{0}),$$$$(q_{1},\sigma _{2}\ldots \sigma _{n},$$$$ Z_{1}\gamma _{1}))\cdot \vdash _{\mathcal {M}}((q_{1},\sigma _{2}\ldots \sigma _{n},Z_{1}\gamma _{1}), (q_{2},\sigma _{3}\ldots \sigma _{n},Z_{2}\gamma _{2}))\ldots \vdash _{\mathcal {M}}((q_{n-1},\sigma _{n},Z_{n-1}\gamma _{n-1}), (q_{n},\varepsilon ,\gamma _{n-1}))\cdot F(q_{n})|\omega =\sigma _{1}\ldots \sigma _{n},$$$$\sigma _{i}\in \Sigma \cup \{\varepsilon \}$$, $$i=1,\ldots ,n$$, $$(q,q_{1},\ldots ,q_{n})\in Q^{n+1}$$, $$Z_{1},\ldots ,Z_{n-1}\in \Gamma $$, $$\gamma _{1},\ldots ,\gamma _{n-1}\in \Gamma ^{*}\}=\langle \mathcal {M}\rangle _{r}(\omega )$$.

(2) implies (1): Let *f* be *r*-recognizable by a WPDA$$^{\emptyset }$$$$\mathcal {N}=(Q, \Sigma ,\Gamma ,\delta ,I,Z_{0},\emptyset )$$ over *A*. Then we construct a WPDA $$\mathcal {M}=(Q^{'},\Sigma , \Gamma ^{'},\eta ,I^{'},X_{0},F)$$ as follows: $$Q^{'}=Q\cup \{q_{0},q_{f}\}$$, $$q_{0},q_{f}\notin Q$$; $$\Gamma ^{'}=\Gamma \cup \{X_{0}\}$$, $$X_{0}\notin \Gamma $$; $$I^{'}:Q^{'}\rightarrow A$$ is a mapping defined by letting $$I^{'}(q_{0})=1$$, $$I^{'}(q_{f})=0$$ and $$I^{'}(q)=0$$ if $$q\in Q$$; $$F: Q^{'}\rightarrow A$$ is a mapping defined by letting $$F(q_{f})=1$$ and otherwise $$F(q)=0$$; $$\eta :Q^{'}\times (\Sigma \cup \{\varepsilon \})\times \Gamma ^{'}\times Q^{'}\times \Gamma ^{'*}\rightarrow A$$ is a mapping defined by

(i) $$\eta (q_{0},\varepsilon ,X_{0},q,Z_{0}X_{0})=I(q)$$, $$\eta (q,\varepsilon ,X_{0},q_{f},\varepsilon )=1$$, $$\forall q\in Q$$;
(ii) $$\eta (q,\tau ,Z,p,\gamma )=\delta (q,\tau ,Z,p,\gamma )$$, $$\forall q,p\in Q$$, $$\tau \in \Sigma \cup \{\varepsilon \}$$, $$Z\in \Gamma $$, $$\gamma \in \Gamma ^{*}$$;
(iii) Otherwise, $$\eta (q^{'},\tau ^{'},Z^{'},p^{'},\gamma ^{'})=0$$.

Next we prove $$\langle \mathrm{rec}$$$$(\mathcal {N})\rangle _{r}\!=\!\langle \mathcal {M}\rangle _{r}$$. In fact, for every $$\omega \!\in \!\Sigma ^{*}$$, we have $$\langle \mathrm{rec} (\mathcal {N})\rangle _{r}(\omega )=\sum \{I(q)\cdot \vdash _{\mathcal {N}}((q,\omega ,Z_{0}),(q_{1},\sigma _{2}\ldots \sigma _{n},$$$$ Z_{1}\gamma _{1}))\cdot \vdash _{\mathcal {N}}((q_{1},\sigma _{2}\ldots \sigma _{n},$$$$Z_{1}\gamma _{1}), (q_{2},\sigma _{3}\ldots \sigma _{n},Z_{2}\gamma _{2}))\ldots \vdash _{\mathcal {N}}((q_{n-1},\sigma _{n},Z_{n-1}\gamma _{n-1}), (q_{n},\varepsilon ,\varepsilon ))|\omega =\sigma _{1}\ldots \sigma _{n},$$$$\sigma _{i}\in \Sigma \cup \{\varepsilon \}$$, $$i=1,\ldots ,n, (q,q_{1},\ldots ,q_{n})\!\in \! Q^{n+1}$$, $$Z_{1},\ldots ,Z_{n-1}\!\in \!\Gamma $$, $$\gamma _{1},\ldots ,\gamma _{n-1}\!\in \!\Gamma ^{*}\} =\sum \{I^{'}(q_{0})\cdot \vdash _{\mathcal {M}}((q_{0},\omega ,X_{0}),(q,\omega ,Z_{0}X_{0})) \cdot \vdash _{\mathcal {M}}((q,\omega ,Z_{0}X_{0}),(q_{1},\sigma _{2}\ldots $$$$\sigma _{n}, Z_{1}\gamma _{1}X_{0}))\cdot \vdash _{\mathcal {M}}((q_{1},\sigma _{2}\ldots \sigma _{n}, Z_{1}\gamma _{1}X_{0}), (q_{2},\sigma _{3}\ldots \sigma _{n},$$$$Z_{2}\gamma _{2}X_{0}))\ldots \vdash _{\mathcal {M}}((q_{n-1},\sigma _{n},$$$$Z_{n-1}\gamma _{n-1}X_{0}), (q_{n},\varepsilon ,X_{0}))\cdot $$$$\vdash _{\mathcal {M}}((q_{n},\varepsilon ,X_{0}), (q_{f},\varepsilon ,\varepsilon ))\cdot F(q_{f})|\omega =\sigma _{1}\ldots \sigma _{n},$$$$\sigma _{i}\in \Sigma \cup \{\varepsilon \}$$, $$i=1,\ldots ,n$$, $$(q,q_{1},\ldots ,q_{n})\in Q^{n+1}$$, $$Z_{1},\ldots ,$$$$Z_{n-1}\in \Gamma $$, $$\gamma _{1},\ldots ,\gamma _{n-1}\in \Gamma ^{*}\}=\langle \mathcal {M}\rangle _{r}(\omega )$$.

Next we give a simple characterization when for every WPDA or WPDA$^{\emptyset }$ over *A*, its run semantics and breadth-first semantics coincide.

### **Proposition 3**

*LetAbe a completely idempotent strong bimonoid. Then the following statements are equivalent:*

1. *Ais left and right distributive;*
2. $\langle \mathcal {M}\rangle _{r}=\langle \mathcal {M}\rangle _{b}$*for every WPDA*

$$\mathcal {M}=(Q, \Sigma ,\Gamma ,\delta ,I,Z_{0},F)$$*overAwith*$$\delta (q,\varepsilon ,Z,q,Z)\in \{0,1\}$$*for*$$q\in Q$$, $$Z\in \Gamma $$.

### *Proof*

(1) implies (2): Obviously.

(2) implies (1): Let $\langle \mathcal {M}\rangle _{r}=\langle \mathcal {M}\rangle _{b}$ for every WPDA $\mathcal {M}$ over *A*. Let $a,m,c\in A$. Then we construct a WPDA $\mathcal {M}=(Q, \Sigma ,\Gamma ,\delta ,I,Z_{0},F)$ over *A* as follows:

$$Q=\{q_{0},q_{1},q_{2},q_{3}\}$$, $$\Sigma =\{\sigma \}$$, $$\Gamma =\{Z_{0}\}$$, $$I(q_{0})=1,$$$$I(q_{1})=I(q_{2})=I(q_{3})=0$$, $$F(q_{3})=c$$, $$F(q_{0})=F(q_{1})=F(q_{2})=0$$, $$\delta (q_{0},\sigma ,Z_{0},q_{1},Z_{0})=a$$, $$\delta (q_{0},\sigma ,Z_{0},q_{2},Z_{0})=m$$, $$\delta (q_{1},\sigma ,Z_{0},q_{3},Z_{0})=1$$, $$\delta (q_{2},\sigma ,Z_{0},q_{3},Z_{0})=1$$, and otherwise $$\delta (p,\tau ,Z_{0},q,\gamma )=0$$.

Since $$\langle \mathcal {M}\rangle _{r}=\langle \mathcal {M}\rangle _{b}$$, $$\langle \mathcal {M}\rangle _{r}(\sigma \sigma )=I(q_{0})\cdot \vdash _{\mathcal {M}}((q_{0},\sigma \sigma ,Z_{0}),(q_{1},\sigma ,Z_{0})) \cdot \vdash _{\mathcal {M}}((q_{1},\sigma ,Z_{0}),(q_{3},\varepsilon ,Z_{0}))\cdot F(q_{3})$$$$+I(q_{0})\cdot \vdash _{\mathcal {M}}((q_{0},\sigma \sigma ,Z_{0}),$$$$(q_{2},\sigma ,Z_{0}))$$$$ \cdot \vdash _{\mathcal {M}}((q_{2},\sigma ,Z_{0}),(q_{3},\varepsilon ,Z_{0}))\cdot F(q_{3})$$$$=1\cdot a\cdot 1\cdot c+1\cdot m\cdot 1\cdot c=a\cdot c+m\cdot c$$, and $$\langle \mathcal {M}\rangle _{b}(\sigma \sigma )=I(q_{0})\cdot (\vdash _{\mathcal {M}}((q_{0},\sigma \sigma ,Z_{0}),(q_{1},\sigma ,Z_{0})) \cdot \vdash _{\mathcal {M}}((q_{1},\sigma ,Z_{0}),(q_{3},\varepsilon ,Z_{0}))$$$$+\vdash _{\mathcal {M}}((q_{0},\sigma \sigma ,Z_{0}),(q_{2},\sigma ,Z_{0})) \cdot \vdash _{\mathcal {M}}((q_{2},\sigma ,Z_{0}),(q_{3},\varepsilon ,Z_{0})))\cdot F(q_{3})$$$$=1\cdot (a\cdot 1+ m\cdot 1)\cdot c=(a+m)\cdot c$$,

we have $$a\cdot c+m\cdot c=(a+m)\cdot c$$, $$\forall a,m,c\in A$$.

Construct a WPDA $\mathcal {M}^{'}=(Q^{'}, \Sigma ,\Gamma ,\eta ,I^{'},Z_{0},F^{'})$ over *A* as follows:

$$Q^{'}=\{p_{0},p_{1},p_{2},p_{3}\}$$, $$\Sigma =\{\sigma \}$$, $$\Gamma =\{Z_{0}\}$$, $$I^{'}(p_{0})=c,$$$$I^{'}(p_{1})=I^{'}(p_{2})=I^{'}(p_{3})=0$$, $$F^{'}(p_{3})=1$$, $$F^{'}(p_{0})=F^{'}(p_{1})=F^{'}(p_{2})=0$$, $$\eta (p_{0},\sigma ,Z_{0},p_{1},Z_{0})=a$$, $$\eta (p_{0},\sigma ,Z_{0},$$$$p_{2},Z_{0})=m$$, $$\eta (p_{1},\sigma ,Z_{0},p_{3},Z_{0})=1$$, $$\eta (p_{2},\sigma ,Z_{0},p_{3},Z_{0})=1$$, and otherwise $$\eta (p,\tau ,Z_{0},q,\gamma )=0$$. Then $$\langle \mathcal {M}^{'}\rangle _{r}=\langle \mathcal {M}^{'}\rangle _{b}$$.

Since $$\langle \mathcal {M}^{'}\rangle _{r}(\sigma \sigma )=I^{'}(p_{0})\cdot \vdash _{\mathcal {M}^{'}}((p_{0},\sigma \sigma ,Z_{0}),(p_{1},\sigma ,Z_{0})) \cdot \vdash _{\mathcal {M}^{'}}((p_{1},\sigma ,Z_{0}),(p_{3},\varepsilon ,Z_{0}))\cdot F^{'}(p_{3})$$$$+I^{'}(p_{0})\cdot \vdash _{\mathcal {M}^{'}}((p_{0}, \sigma \sigma , Z_{0}),$$$$(p_{2},\sigma ,Z_{0}))$$$$ \cdot \vdash _{\mathcal {M}^{'}}((p_{2},\sigma ,Z_{0}),(p_{3},\varepsilon ,Z_{0}))\cdot F^{'}(p_{3})$$$$=c\cdot a\cdot 1\cdot 1+c\cdot m\cdot 1\cdot 1=c\cdot a+c\cdot m$$ and $$\langle \mathcal {M}^{'}\rangle _{b}(\sigma \sigma )=I^{'}(p_{0})\cdot (\vdash _{\mathcal {M}^{'}}((p_{0},\sigma \sigma ,Z_{0}),(p_{1},\sigma ,Z_{0})) \cdot \vdash _{\mathcal {M}^{'}}((p_{1},\sigma ,Z_{0}),(p_{3},\varepsilon ,Z_{0}))$$$$+\vdash _{\mathcal {M}^{'}}((p_{0},\sigma \sigma ,Z_{0}),$$$$(p_{2},\sigma , Z_{0}))$$$$\cdot \vdash _{\mathcal {M}^{'}}((p_{2},\sigma ,Z_{0}),(p_{3},\varepsilon ,Z_{0})))\cdot F^{'}(p_{3})$$$$=c\cdot (a\cdot 1+m\cdot 1)\cdot 1=c\cdot (a+m)$$, we have $$c\cdot a+c\cdot m=c\cdot (a+m)$$, $$\forall a,m,c\in A$$.

Hence *A* is left and right distributive.

### **Proposition 4**

*LetAbe a completely idempotent strong bimonoid. Then the following statements are equivalent:*

1. *Ais left distributive;*
2. $$\langle \mathrm{rec}(\mathcal {N})\rangle _{r}=\langle \mathrm{rec}(\mathcal {N})\rangle _{b}$$*for every*$$\hbox {WPDA}^{\emptyset }$$$$\mathcal {N}=(Q, \Sigma ,\Gamma ,\delta ,I,Z_{0},F)$$*overAwith*$$\delta (q,\varepsilon ,Z,q,Z)\in \{0,1\}$$*for*$$q\in Q$$, $$Z\in \Gamma $$.

### *Proof*

(1) implies (2): Obviously.

(2) implies (1): Let $$\langle \mathrm{rec}(\mathcal {N})\rangle _{r}=\langle \mathrm{rec}(\mathcal {N})\rangle _{b}$$ for every WPDA$$^{\emptyset }$$$$\mathcal {N}$$ over *A*. Let $$a,m,c\in A$$. Then we construct a WPDA$$^{\emptyset }$$$$\mathcal {N}=(Q, \Sigma ,\Gamma ,\delta ,I,Z_{0},\emptyset )$$ over *A* as follows:

$$Q=\{q_{0},q_{1},q_{2}\}$$, $$\Sigma =\{\sigma \}$$, $$\Gamma =\{Z_{0}\}$$, $$I(q_{0})=c,$$$$I(q_{1})=I(q_{2})=0$$, $$\delta (q_{0},\sigma ,Z_{0},q_{1},\varepsilon )=a$$, $$\delta (q_{0},\sigma ,Z_{0},q_{2},\varepsilon )$$$$=m$$, and otherwise $$\delta (q,\sigma ,Z_{0},p,\gamma )=0$$.

Since $$\langle \mathrm{rec}(\mathcal {N})\rangle _{r}=\langle \mathrm rec(\mathcal {N})\rangle _{b}$$, $$\langle \mathrm{rec}(\mathcal {N})\rangle _{r}(\sigma )=I(q_{0})\cdot \vdash _{\mathcal {N}}((q_{0},\sigma ,Z_{0}),(q_{1},\varepsilon ,\varepsilon ))$$$$+I(q_{0}) \cdot \vdash _{\mathcal {N}}((q_{0},\sigma ,Z_{0}),(q_{2},\varepsilon ,\varepsilon ))$$$$=c\cdot a+c\cdot m$$, and $$\langle \mathrm{rec}(\mathcal {N})\rangle _{b}(\sigma )=I(q_{0})\cdot (\vdash _{\mathcal {N}}((q_{0},\sigma ,Z_{0}),(q_{1},\varepsilon ,\varepsilon ))$$$$+\vdash _{\mathcal {N}}((q_{0},\sigma ,Z_{0}),(q_{2},\varepsilon ,\varepsilon )))$$$$=c\cdot (a+m)$$,

we have $$c\cdot a+c\cdot m=c\cdot (a+m)$$, $$\forall a,m,c\in A$$.

We call a strong bimonoid *A* additively locally finite (multiplicatively locally finite, respectively) if for every finite set $B\subseteq A$, The smallest submonoid of $(A,+,0)$ (of $(A,\cdot ,1)$, respectively) containing *B* is finite. If *A* is both additively and multiplicatively locally finite, then it is called bi-locally finite.

### **Proposition 5**

*Let*$$|\Sigma |\ge 2$$*andAbe a complete strong bimonoid. ThenAis bi-locally finite if and only if*$$Im(\langle \mathcal {M}\rangle _{r})$$*or*$$Im(\langle \mathcal {M}\rangle _{b})$$*is a finite set for every WPDA*$$\mathcal {M}=(Q,\Sigma ,\Gamma ,\delta ,$$$$I,Z_{0},F)$$*overA*.

### *Proof*

Claim 1. If *A* is bi-locally finite, then $$Im(\langle \mathcal {M}\rangle _{r})$$ or $$Im(\langle \mathcal {M}\rangle _{b})$$ is finite for every WPDA $$\mathcal {M}=(Q,\Sigma ,\Gamma ,\delta ,$$$$I,Z_{0},F)$$ over *A*.

In fact, let $T=Im(I)\cup Im(\delta )\cup Im(F)$ and *R* be the submonoid of $(A,\cdot ,1)$ generated by *T*. Then *R* is finite since *T* is finite and $(A,\cdot ,1)$ is multiplicatvely locally finite. Suppose $R^{'}$ is the submonoid of $(A,+,0)$ generated by *R*. Then $R^{'}$ is finite by the additively locally finiteness of $(A,+,0)$. Hence $\langle \mathcal {M}\rangle _{r}(\omega )\in R^{'}$ for every $\omega \in \Sigma ^{*}$ and so $Im(\langle \mathcal {M}\rangle _{r})\subseteq R^{'}$ is finite.

Let $T_{1}$ be the submonoid of $(A,\cdot ,1)$ generated by $Im(\delta )$. Then $T_{1}$ is finite by the multiplicatively locally finiteness of $(A,\cdot ,1)$. Let $T_{2}$ be the submonoid of $(A,+,0)$ generated by $T_{1}$, $R_{1}=\{I(q)\cdot k\cdot F(p)|q,p\in Q, k\in T_{2}\}$ and $T_{3}$ be the submonoid of $(A,+,0)$ generated by $R_{1}$. Then $T_{2},R_{1}$ and $T_{3}$ are finite since *A* is bi-locally finite. Hence $\langle \mathcal {M}\rangle _{b}(\omega )\in T_{3}$ for every $\omega \in \Sigma ^{*}$ and so $Im(\langle \mathcal {M}\rangle _{b})\subseteq T_{3}$ is finite.

Claim 2. If $$Im(\langle \mathcal {M}\rangle _{r})$$ or $$Im(\langle \mathcal {M}\rangle _{b})$$ is finite for every WPDA $$\mathcal {M}=(Q,\Sigma ,\Gamma ,\delta ,$$$$I,Z_{0},F)$$ over *A*, then *A* is bi-locally finite. In fact, it suffices to prove that both the additive monoid $$(A,+,0)$$ and the multiplicative monoid $$(A,\cdot ,1)$$ are locally finite.

For the additive monoid it suffices to prove that for every $$a\in A$$ the cyclic submonoid of $$(A,+,0)$$ generated by *a* is finite because $$+$$ is commutative and associative. Let $$a\in A$$. Then we construct a WPDA $$\mathcal {M}=(Q,\Sigma ,\Gamma ,\delta ,$$$$I,Z_{0},F)$$ over *A* with $$Q=\{p,q\}$$, $$\Gamma =\{Z_{0}\}$$, $$I(p)=1$$, $$I(q)=F(p)=0$$ and $$F(q)=1$$. Moreover, for every $$\tau \in \Sigma $$, $$\delta (p,\tau ,Z_{0},p,Z_{0})=\delta (q,\tau ,Z_{0},q,Z_{0})=1$$, $$\delta (p,\tau ,Z_{0},q,Z_{0})=a$$, and otherwise $$\delta (p_{1},\sigma ,Z_{0},p_{2},\gamma )=0$$. Then for every $$\sigma \in \Sigma $$ and a natural number *n* we have $$\langle \mathcal {M}\rangle _{r}(\sigma ^{n})=\langle \mathcal {M}\rangle _{b}(\sigma ^{n})=a+\cdots +a$$ (*n* times). Thus the finite set $$Im(\langle \mathcal {M}\rangle _{r})\cap Im(\langle \mathcal {M}\rangle _{b})$$ contains the cyclic submonoid of $$(A,+,0)$$ generated by *a*.

Next we prove that the multiplicative monoid $$(A,\cdot ,1)$$ is locally finite. Let *n* be a natural number and $$a_{1},\ldots , a_{n} \in A$$. It suffices to show that the set $$A^{'}=\{a_{l_{1}}\ldots a_{l_{k}}|k$$ is a natural number, $$l_{i}=1,\ldots ,n$$ for $$i=1,\ldots ,k\}$$ is finite. Let $$\tau _{1},\tau _{2}\in \Sigma $$ be distinct symbols. We construct a WPDA $$\mathcal {M}^{'}=(Q^{'},\Sigma ,\Gamma ,\delta ^{'},$$$$I^{'},Z_{0},F^{'})$$ over *A* with $$Q^{'}=\{q_{0},q_{1},\ldots ,q_{n}\}$$, $$I^{'}(q_{0})=F^{'}(q_{0})=1$$ and $$I^{'}(q)=F^{'}(q)=0$$ for every $$q\in Q^{'}{\setminus }\{q_{0}\}$$. The mapping $$\delta ^{'}: Q'\times (\Sigma \cup \{\varepsilon \})\times \Gamma \times Q'\times \Gamma ^{*}\rightarrow A$$ is defined as follows:

$\delta ^{'}(q_{i-1},\tau _{1},Z_{0},q_{i},Z_{0})=1$ and $\delta ^{'}(q_{i},\tau _{2},Z_{0},q_{0},Z_{0})=a_{i}$ for every $i=1,\ldots ,n$; and $\delta ^{'}(q,\sigma ,Z_{0},q^{'},Z_{0})=0$ for every other combination of $\sigma \in \Sigma \cup \{\varepsilon \}$ and $q,q^{'}\in Q^{'}$. Then $\langle \mathcal {M}\rangle _{r}(\tau _{1}^{l_{1}}\tau _{2}\tau _{1}^{l_{2}}\tau _{2}\ldots \tau _{1}^{l_{k}}\tau _{2})=\langle \mathcal {M}\rangle _{b}(\tau _{1}^{l_{1}}\tau _{2}\tau _{1}^{l_{2}}\tau _{2}\ldots \tau _{1}^{l_{k}}\tau _{2})=a_{l_{1}}\ldots a_{l_{k}}$ for every natural number *k* and $l_{i}=1,\ldots ,n$ with $i=1,\ldots ,k$. Thus, $A^{'}\subseteq Im(\langle \mathcal {M}\rangle _{r})\cap Im(\langle \mathcal {M}\rangle _{b})$, and therefore $A^{'}$ is finite.

Next we introduce crisp-simple weighted pushdown automata over complete strong bimonoids.

### **Definition 4**

Let $\mathcal {M}=(Q, \Sigma ,\Gamma ,\delta ,I,Z_{0},F)$ be a WPDA over a complete strong bimonoid *A*. Then $\mathcal {M}$ is called crisp-simple if $Im(I)\cup Im(\delta )\subseteq \{0,1\}$.

### **Proposition 6**

*If a complete strong bimonoidAis multiplicatively locally finite, then for every WPDA overAthere is ar*-*equivalent crisp-simple weighted pushdown automaton overA*.

### *Proof*

Let $$\mathcal {M}=(Q, \Sigma ,\Gamma ,\delta ,I,Z_{0},F)$$ be a WPDA over a complete strong bimonoid *A*. Let $$T=Im(I)\cup Im(\delta )$$ and *R* be the submonoid of $$(A,\cdot ,1)$$ generated by *T*. Then *R* is finite since *T* is finite and *A* is multiplicatively locally finite. We construct a crisp-simple weighted pushdown automaton over *A*, $$\mathcal {M}^{'}=(Q^{'},\Sigma ,\Gamma ,\delta ^{'},I^{'},Z_{0},F^{'})$$, where $$Q^{'}=Q\times (R{\setminus }\{0\})$$, $$I^{'}{:}\;Q^{'}\rightarrow A$$ is defined by $$I^{'}((q,k))=1$$ when $$k=I(q)\ne 0$$ and otherwise $$I^{'}((q,k))=0$$, and $$F^{'}{:}\;Q^{'}\rightarrow A$$ is defined by $$F^{'}((q,k))=k\cdot F(q)$$ for every $$(q,k)\in Q^{'}$$. The mapping $$\delta ^{'}{:}\;Q^{'}\times (\Sigma \cup \{\varepsilon \})\times \Gamma \times Q^{'}\times \Gamma ^{*}\rightarrow \{0,1\}$$ is given by $$\delta ^{'}((q,k),\tau ,X,(p,l), \gamma )=1$$ for every $$(q,k)\in Q^{'}$$, $$\tau \in \Sigma \cup \{\varepsilon \}$$, $$X\in \Gamma $$ and $$((p,l),\gamma )\in B$$, $$\delta ^{'}((q,k),\tau ,X,(p,l),\gamma )=0$$ for every $$(q,k)\in Q^{'}$$, $$\tau \in \Sigma \cup \{\varepsilon \}$$, $$X\in \Gamma $$ and $$((p,l),\gamma )\notin B$$, where $$B=\{((q^{'},k\cdot k^{'}),\gamma ^{'})|\delta (q,\tau ,X,q^{'},\gamma ^{'})=k^{'}, q^{'}\in Q, \gamma ^{'}\in \Gamma ^{*},k^{'}\ne 0,k\cdot k^{'}\ne 0\}$$. Then for every $$\omega \in \Sigma ^{*}$$ by letting $$\omega =\sigma _{1}\ldots \sigma _{n}\in \Sigma ^{*}$$, $$\sigma _{i}\in \Sigma \cup \{\varepsilon \}$$ with $$i=1,\ldots ,n$$, we have $$I(q)\cdot \vdash _{\mathcal {M}}((q,\omega ,Z_{0}),(q_{1},\sigma _{2}\ldots \sigma _{n}, Z_{1}\gamma _{1}))\cdot \vdash _{\mathcal {M}}((q_{1},$$$$\sigma _{2}\ldots \sigma _{n}, Z_{1}\gamma _{1}),(q_{2},\sigma _{3}\ldots \sigma _{n},$$$$ Z_{2}\gamma _{2}))\ldots \vdash _{\mathcal {M}}(((q_{n-1},$$$$\sigma _{n}, Z_{n-1}\gamma _{n-1}),(q_{n},\varepsilon , Z_{n}\gamma _{n}))\ne 0$$ if and only if $$I^{'}((q,I(q)))\cdot \vdash _{\mathcal {M}^{'}}(((q,I(q)),\omega ,Z_{0}),((q_{1},a_{1}), \sigma _{2}\ldots $$$$\sigma _{n}, Z_{1}\gamma _{1}))\cdot \vdash _{\mathcal {M}^{'}}((q_{1},a_{1}), \sigma _{2}$$$$\ldots \sigma _{n}, Z_{1}\gamma _{1}),((q_{2},a_{2}),\sigma _{3}\ldots $$$$\sigma _{n}, Z_{2}\gamma _{2}))\ldots \vdash _{\mathcal {M}^{'}}((q_{n-1},a_{n-1}),\sigma _{n}, Z_{n-1}\gamma _{n-1}),((q_{n},$$$$a_{n}),\varepsilon , Z_{n}\gamma _{n}))=1$$, where $$q,q_{1},\ldots ,q_{n}\in Q$$; $$Z_{1},Z_{2},\ldots ,Z_{n-1}\in \Gamma $$; $$\gamma _{1},\ldots , \gamma _{n}\in \Gamma ^{*}$$; $$Z_{n}\in \Gamma ^{*}$$, $$a_{1}=I(q)\cdot \vdash _{\mathcal {M}}((q,\omega ,Z_{0}),(q_{1},\sigma _{2}\ldots \sigma _{n}, Z_{1}\gamma _{1}))$$, $$a_{j}=a_{j-1}\cdot \vdash _{\mathcal {M}}((q_{j-1},\sigma _{j}\ldots \sigma _{n}, Z_{j-1}\gamma _{j-1}),$$$$(q_{j},\sigma _{j+1} \ldots \sigma _{n}, Z_{j}\gamma _{j}))$$, and $$a_{n}=a_{n-1}\cdot \vdash _{\mathcal {M}}((q_{n-1},\sigma _{n}, Z_{n-1}\gamma _{n-1}),$$$$(q_{n},\varepsilon , Z_{n}\gamma _{n}))$$, $$j=2,\ldots ,n-1$$. Hence $$\langle \mathcal {M}\rangle _{r}(\omega )=\sum \{I(q)\cdot \vdash _{\mathcal {M}}((q,\omega ,Z_{0}),(q_{1},\sigma _{2}\ldots \sigma _{n},$$$$ Z_{1}\gamma _{1}))\cdot \vdash _{\mathcal {M}}((q_{1},\sigma _{2}\ldots \sigma _{n},$$$$ Z_{1}\gamma _{1}), (q_{2},\sigma _{3}\ldots \sigma _{n}, Z_{2}\gamma _{2}))\ldots \vdash _{\mathcal {M}}(((q_{n-1},\sigma _{n}, Z_{n-1}\gamma _{n-1}),(q_{n},\varepsilon , Z_{n}\gamma _{n}))\cdot F(q_{n})|(q,$$$$q_{1},\ldots ,q_{n})\in Q^{n+1}, Z_{1},\ldots ,Z_{n-1}\!\in \!\Gamma ,\gamma _{1},\ldots ,\gamma _{n}\!\in \!\Gamma ^{*},$$$$ Z_{n}\in \Gamma ^{*}\}$$$$=\sum \{F^{'}((q_{n},a_{n}))|a_{n}$$$$=I(q)\cdot \vdash _{\mathcal {M}}((q,\omega ,Z_{0}),$$$$(q_{1},\sigma _{2}\ldots \sigma _{n}, Z_{1}\gamma _{1}))\cdot \vdash _{\mathcal {M}}((q_{1},\sigma _{2}\ldots \sigma _{n}, Z_{1}\gamma _{1}), (q_{2},\sigma _{3}\ldots $$$$\sigma _{n}, Z_{2}\gamma _{2}))\ldots \vdash _{\mathcal {M}}(((q_{n-1},\sigma _{n}, Z_{n-1}\gamma _{n-1}), (q_{n},\varepsilon , Z_{n}\gamma _{n}))$$$$\ne 0,(q,q_{1},\ldots ,q_{n})\!\in \! Q^{n+1}, Z_{1},\ldots ,Z_{n-1}\!\in \!\Gamma ,\gamma _{1},\ldots ,\gamma _{n}\in \Gamma ^{*}, Z_{n}\in \Gamma ^{*}\}$$$$=\sum \{I^{'}((q,I(q)))\cdot \vdash _{\mathcal {M}^{'}}(((q,I(q)),\omega ,Z_{0}),$$$$((q_{1},a_{1}), \sigma _{2}\ldots \sigma _{n}, Z_{1}\gamma _{1}))\cdot \vdash _{\mathcal {M}^{'}}((q_{1},a_{1}), \sigma _{2}\ldots \sigma _{n}, Z_{1}\gamma _{1}),$$$$((q_{2},a_{2}),$$$$\sigma _{3}\ldots \sigma _{n}, Z_{2}\gamma _{2}))\ldots \vdash _{\mathcal {M}^{'}}((q_{n-1},a_{n-1}),\sigma _{n},$$$$ Z_{n-1}\gamma _{n-1}),((q_{n},a_{n}),\varepsilon , Z_{n}\gamma _{n}))\cdot F^{'}((q_{n},a_{n}))$$$$|a_{1}=I(q)\cdot \vdash _{\mathcal {M}}((q,\omega ,Z_{0}),(q_{1},\sigma _{2}\ldots \sigma _{n}, Z_{1}\gamma _{1}))$$, $$a_{j}=a_{j-1}\cdot \vdash _{\mathcal {M}}((q_{j-1},$$$$\sigma _{j}\ldots \sigma _{n}, Z_{j-1}\gamma _{j-1}),(q_{j}, \sigma _{j+1}\ldots \sigma _{n}, Z_{j}\gamma _{j}))$$, $$j=2,\ldots ,n-1$$, $$a_{n}=a_{n-1}\cdot \vdash _{\mathcal {M}}((q_{n-1},\sigma _{n}, Z_{n-1}\gamma _{n-1}),$$$$(q_{n},\varepsilon , Z_{n}\gamma _{n}))$$, $$q,q_{1},\ldots ,q_{n}\in Q$$, $$Z_{1},Z_{2},\ldots ,Z_{n-1}\in \Gamma $$, $$\gamma _{1},\ldots ,\gamma _{n}\in \Gamma ^{*}$$, $$Z_{n}\in \Gamma ^{*}\}=\langle \mathcal {M'}\rangle _{r}(\omega )$$.

So $$\langle \mathcal {M}\rangle _{r}=\langle \mathcal {M'}\rangle _{r}$$.

Clearly a crisp-simple weighted pushdown automaton over a complete strong bimonoid *A* is also a WPDA by Definition 4. So Proposition 4 also shows that WPDAs over a complete strong bimonoid *A* and crisp-simple weighted pushdown automata over *A* are r-equivalent if *A* is multiplicatively locally finite.

## WCFGs over complete strong bimonoids

It is well known that fuzzy grammars have become a necessary tool for the analysis of fuzzy finite automata. In this section, we will introduce the definition of weighted context-free grammars over complete strong bimonoids and investigate the relationship between weighted context-free grammars and weighted pushdown automata over complete strong bimonoids.

### **Definition 5**

Let *A* be a complete strong bimonoid. Then a weighted context-free grammar (WCFG for short) over *A* is a system $G=(N,T,P,I)$, where

(i) *N* is a finite nonempty alphabet of variables;
(ii) *T* is a finite nonempty alphabet of terminals and $T\cap N=\emptyset$;
(iii) $I{:}\;N\rightarrow A$ is a mapping called the weighted subset of initial symbols;
(iv) *P* is a finite collection of productions and $P=\{u\overset{r}{\rightarrow } \upsilon |u\in N, \upsilon \in (N\cup T)^{*},\rho (u\rightarrow \upsilon )=r\in A{\setminus }\{0\}\}$, where $\rho$ is a mapping from $(N\cup T)^{*}\times (N\cup T)^{*}$ to *A*, $\rho (u,\upsilon )$ means the membership degree that *u* will be replaced by $\upsilon$, denoted by $\rho (u,\upsilon )=\rho (u\rightarrow \upsilon )$.

For the sake of convenience, $u\overset{r}{\rightarrow }\upsilon$ is sometimes abbreviated as $u\rightarrow \upsilon$ in *P*. For $\alpha ,\beta \in (N\cup T)^{*}$, if $\alpha \upsilon \beta$ is directly derivable from $\alpha u\beta$, i.e., $\alpha u\beta \Rightarrow \alpha \upsilon \beta$ by the production $u\rightarrow \upsilon$ in *P*, then we define $\rho (\alpha u\beta \Rightarrow \alpha \upsilon \beta )=\rho (u\rightarrow \upsilon )$. If $\omega _{i}\in (N\cup T)^{*}$ for $i=1,\ldots ,n$, and $\omega _{i+1}$ is directly derivable from $\omega _{i}$ for $i=1,\ldots ,n-1$, then we say that $\omega _{n}$ is derivable from $\omega _{1}$ and call $\omega _{1}\Rightarrow \omega _{2}\Rightarrow \cdots \Rightarrow \omega _{n}$ the derivation chain *c* of $\omega _{n}$ (from $\omega _{1}$), which is written as, $\omega _{1}\Rightarrow _{c}^{*}\omega _{n}$. And we define $\rho (\omega _{1}\Rightarrow _{c}^{*}\omega _{n})= \rho (\omega _{1}\Rightarrow \omega _{2})\ldots \rho (\omega _{i}\Rightarrow \omega _{i+1})\ldots \rho (\omega _{n-1}\Rightarrow \omega _{n})$. Moreover, for the derivation chain $\omega _{1}\Rightarrow \omega _{2}\Rightarrow \cdots \Rightarrow \omega _{n}$ and for any $i\in \{1,2,\ldots ,n-1\}$, if only the leftmost variable in $\omega _{i}$ is replaced in the process of $\omega _{i}\Rightarrow \omega _{i+1}$, then $\omega _{1}\Rightarrow \omega _{2}\Rightarrow \cdots \Rightarrow \omega _{n}$ is called the leftmost derivation chain, which will be abbreviated as $\omega _{1}\Rightarrow ^{L}\omega _{2}\Rightarrow ^{L}\cdots \Rightarrow ^{L}\omega _{n}$.

Based on run semantics, the formal power series $\langle G\rangle _{r}: T^{*}\rightarrow A$ generated by WCFG $G=(N,T,P,I)$ is defined by, for every $\theta =\omega _{n}\in T^{*}$ and $n\ge 1$,

$$\langle G\rangle _{r}(\theta )=\sum \{I(\omega _{0})\cdot \rho (\omega _{0}\Rightarrow \omega _{1}) \ldots \rho (\omega _{n-1}\Rightarrow \omega _{n}) |\omega _{0}\in N, \omega _{1},\ldots , \omega _{n-1}\in (N\cup T)^{*}\}$$.

Based on breadth-first algebraic semantics, the formal power series $\langle G\rangle _{b}: T^{*}\rightarrow A$ generated by WCFG $G=(N,T,P,I)$ is defined by, for every $\theta =\omega _{n}\in T^{*}$ and $n\ge 1$, $\langle G\rangle _{b}(\theta )=\sum \{I(\omega _{0}) \cdot (\sum \{\rho (\omega _{0}\Rightarrow \omega _{1}) \ldots \rho (\omega _{n-1}\Rightarrow \omega _{n}) | \omega _{1},\ldots , \omega _{n-1}\in (N\cup T)^{*}\})|\omega _{0}\in N\}$.

Two WCFGs $G_{1}$ and $G_{2}$ are said to be *x*-equivalent provided that they generate the same formal power series, that is, $\langle G_{1}\rangle _{x}=\langle G_{2}\rangle _{x}$ for $x\in \{r,b\}$.

### **Proposition 7**

*Let*$G=(N,T,P,I)$*be a WCFG over a complete and idempotent strong bimonoidAandAsatisfy the following conditions:*

$$\begin{aligned} a\cdot b=b\cdot a,\quad \forall a,\ b \in A, \end{aligned}$$

*Then*$\langle G\rangle _{x}^{L}=\langle G\rangle _{x}$*for*$x\in \{r,b\}$, *where*$\langle G\rangle _{r}^{L}(\theta )=\sum \{I(\omega _{0})\cdot \rho (\omega _{0}\Rightarrow ^{L}\omega _{1})\cdot \rho (\omega _{1}\Rightarrow ^{L}\omega _{2})\ldots \rho (\omega _{n-1}\Rightarrow ^{L}\omega _{n})|\omega _{0}\in N, \omega _{1},\ldots ,\omega _{n-1}\in (N\cup T)^{*}\}$, *and*$\langle G\rangle _{b}^{L}(\theta )=\sum \{I(\omega _{0})\cdot (\sum \{ \rho (\omega _{0}\Rightarrow ^{L}\omega _{1})\cdot \rho (\omega _{1}\Rightarrow ^{L}\omega _{2})\ldots \rho (\omega _{n-1}\Rightarrow ^{L}\omega _{n})|\omega _{1},\ldots ,\omega _{n-1}\in (N\cup T)^{*}\})|\omega _{0}\in N\}$, $\forall \omega _{n}=\theta \in T^{*}, n\ge 1$.

### *Proof*

It suffices to prove the following statement by induction on the step number *n* of derivation: there exists the corresponding leftmost derivation chain such that,

$$\begin{aligned} \rho (\omega _{0}\Rightarrow ^{*L}\theta )=\rho (\omega _{0}\Rightarrow ^{*}\theta ),\ \forall \omega _{0}\Rightarrow ^{*}\theta ,\ \theta \in T^{*}. \end{aligned}$$

In fact, if $$n=1$$, then $$\omega _{0}\Rightarrow \theta $$ is the leftmost derivation and so the above statement holds. Suppose the statement is true when the step number of derivation satisfies the condition that $$n< k$$, where *k* is a positive integer. Then, for any derivation chain associated with step number $$n=k$$, $$\omega _{0}\Rightarrow ^{*}\theta ,\theta \in T^{*}$$, that is, $$\omega _{0}\Rightarrow X_{1}X_{2}\ldots X_{m}\Rightarrow ^{*}\alpha _{1}X_{2}\ldots X_{m}\Rightarrow ^{*}\ldots \Rightarrow ^{*}\alpha _{1}\alpha _{2}\ldots \alpha _{m-1}X_{m} \Rightarrow ^{*}\alpha _{1}\alpha _{2}\ldots $$$$\alpha _{m-1}\alpha _{m}$$, where $$\theta =\alpha _{1}\alpha _{2}\ldots \alpha _{m-1}\alpha _{m}$$, $$\rho (\omega _{0}\Rightarrow ^{*}\theta )=\rho (\omega _{0}\Rightarrow X_{1}X_{2} \ldots X_{m})\cdot \rho (X_{1}X_{2}\ldots X_{m}\Rightarrow ^{*}\alpha _{1}X_{2}\ldots X_{m})\ldots \rho (\alpha _{1}\alpha _{2}\ldots X_{m-1}X_{m}\Rightarrow ^{*} \alpha _{1}\alpha _{2}\ldots \alpha _{m-1}X_{m})\cdot \rho (\alpha _{1}\alpha _{2}\ldots \alpha _{m-1}X_{m}\Rightarrow ^{*} \alpha _{1}\alpha _{2}\ldots \alpha _{m-1}\alpha _{m})$$. If $$X_{i}=\alpha _{i}$$, then $$X_{i}$$ is obtained from the first step of derivation. Otherwise, if $$X_{i}\ne \alpha _{i}$$, then the derivation step number of $$X_{i}\Rightarrow ^{*}\alpha _{i}$$ is not more than $$n-1$$. By induction, there exists the leftmost derivation $$X_{i}\Rightarrow ^{*L}\alpha _{i}$$ such that $$\rho (X_{i}\Rightarrow ^{*}\alpha _{i})=\rho (X_{i}\Rightarrow ^{*L}\alpha _{i})$$.

Hence $$\omega _{0}\Rightarrow ^{L} X_{1}X_{2}\ldots X_{m}\Rightarrow ^{*L}\alpha _{1}X_{2}\ldots X_{m}\Rightarrow ^{*L}\cdots \Rightarrow ^{*L}\alpha _{1}\alpha _{2}\ldots \alpha _{m-1}X_{m} \Rightarrow ^{*L}\alpha _{1}\alpha _{2}\ldots \alpha _{m-1}\alpha _{m}$$.

Since $$a\cdot b=b\cdot a,\quad \forall a,\ b \in A,$$$$\rho (\omega _{0}\Rightarrow ^{*}\theta )=\rho (\omega _{0}\Rightarrow X_{1}X_{2}\ldots X_{m})\cdot \rho (X_{1}X_{2}\ldots X_{m}\Rightarrow ^{*}\alpha _{1}X_{2}\ldots X_{m})\ldots \rho (\alpha _{1}\alpha _{2}\ldots X_{m-1}X_{m}\Rightarrow ^{*} \alpha _{1}\alpha _{2}\ldots \alpha _{m-1} X_{m})\cdot \rho (\alpha _{1}\alpha _{2}\ldots \alpha _{m-1}X_{m}\Rightarrow ^{*} \alpha _{1}\alpha _{2}\ldots \alpha _{m-1}\alpha _{m})= \rho (\omega _{0}\Rightarrow ^{L} X_{1}X_{2}\ldots X_{m})\cdot \rho (X_{1}X_{2}\ldots X_{m}\Rightarrow ^{*L}\alpha _{1}X_{2}\ldots X_{m})\ldots \rho (\alpha _{1}\alpha _{2}\ldots X_{m-1}X_{m}\Rightarrow ^{*L} \alpha _{1}\alpha _{2}\ldots \alpha _{m-1}X_{m})\cdot \rho (\alpha _{1}\alpha _{2}\ldots \alpha _{m-1}X_{m}\Rightarrow ^{*L} \alpha _{1}\alpha _{2}\ldots \alpha _{m-1}\alpha _{m})= \rho (\omega _{0}\Rightarrow ^{*L}\theta )$$.

Since $a+a=a,\ \forall a,\ b \in A,$ it follows by definition that $\langle G\rangle _{x}^{L}=\langle G\rangle _{x}$, $\forall x\in \{r,b\}$.

Based on breadth-first algebraic semantics and run semantics, we will investigate the relationship between weighted context-free grammars and weighted pushdown automata over complete strong bimonoids. For a formal power series generated by a WCFG, how to construct a weighted pushdown automaton over a complete strong bimonoid to recognize it? And for a formal power series recognized by a weighted pushdown automaton over a complete strong bimonoid, how to construct a WCFG to generate it? Next We will solve the above problems.

### **Proposition 8**

*Let*$$G=(N,T,P,I)$$*be a WCFG over a complete strong bimonoidA*. *Then there exists a WPDA*$$^{\emptyset }$$$$\mathcal {M}=(Q,\Sigma ,\Gamma ,\delta ,\sigma ,Z_{0},\emptyset )$$*overAsuch that*

$$\begin{aligned} \langle rec(\mathcal {M})\rangle _{x}=\langle G\rangle _{x}^{L},\quad \forall x\in \{r,b\}. \end{aligned}$$

### *Proof*

Let $$G=(N,T,P,I)$$ be a WCFG over a complete strong bimonoid *A*. Then a WPDA$$^{\emptyset }$$$$\mathcal {M}=(Q,\Sigma ,\Gamma ,\delta ,\sigma ,Z_{0},\emptyset )$$ over *A* could be constructed as follows: $$Q=N\cup \{q\},q\notin N$$, $$\Sigma =T$$, $$\Gamma =N\cup T\cup \{Z_{0}\}$$, $$Z_{0}\notin N\cup T$$, the mapping $$\delta : Q\times (\Sigma \cup \{\varepsilon \})\times \Gamma \times Q\times \Gamma ^{*}\longrightarrow A$$ is defined by,

1. $$\delta (q,\varepsilon ,X,q,\gamma )=\rho (X\rightarrow \gamma )$$ whenever $$X\rightarrow \gamma \in P$$;
2. $$\delta (q,a,a,q,\varepsilon )=1$$ whenever $$a\in T$$;
3. $$\delta (p,\varepsilon ,Z_{0},q,p)=1$$ whenever $$p\in N$$;
4. otherwise, $$\delta (q_{1},u,X,q_{2},\gamma )=0$$.

The mapping $\sigma : Q\rightarrow A$ is given by $\sigma (p)=I(p),\forall p\in N$, and $\sigma (q)=0$.

Noting that every nonterminal string may be remarked as $\alpha X\gamma$, where $\alpha \in T^{*}, X\in N, \gamma \in \Gamma ^{*}$. Then the elements of *P* must be the following forms:

$$Y\rightarrow \alpha X\gamma $$ or $$Y\rightarrow \alpha \alpha _{1}, \alpha \in T^{*}, \alpha _{1}\in T^{*}, Y,X\in N, \gamma \in \Gamma ^{*}.$$

Clearly, $$\forall \omega \in T^{*}$$, $$\forall S,X,X_{1},\ldots , X_{n}\in N, \alpha ,\alpha _{1},\ldots ,\alpha _{n}\in T^{*},\gamma ,\gamma _{1},\ldots ,\gamma _{n}\in \Gamma ^{*}$$, $$S\Rightarrow \alpha X\gamma \Rightarrow ^{L}\alpha \alpha _{1}X_{1}\gamma _{1}\Rightarrow ^{L} \alpha \alpha _{1}\alpha _{2}X_{2}\gamma _{2}\Rightarrow ^{L}\cdots \Rightarrow ^{L} \alpha \alpha _{1}\ldots \alpha _{n}X_{n}\gamma _{n}\Rightarrow ^{L}\omega $$, where $$\rho (S\Rightarrow \alpha X\gamma \Rightarrow ^{L}\alpha \alpha _{1}X_{1}\gamma _{1}\Rightarrow ^{L} \alpha \alpha _{1}\alpha _{2}X_{2}\gamma _{2}\Rightarrow ^{L}\cdots \Rightarrow ^{L} \alpha \alpha _{1}\ldots \alpha _{n}X_{n}\gamma _{n}\Rightarrow ^{L}\omega )=k\in A{\setminus }\{0\}$$; if and only if $$\vdash _{\mathcal {M}}((S,\omega ,Z_{0}),(q,\omega ,S))\cdot \vdash _{\mathcal {M}}((q,\omega ,S),(q,\omega ,\alpha X\gamma ))\cdot \vdash _{\mathcal {M}}^{|\alpha |}((q,\omega ,\alpha X\gamma ),(q,\omega _{1},$$$$X\gamma ))\cdot \vdash _{\mathcal {M}}((q,\omega _{1},X\gamma ),(q,\omega _{1}, \alpha _{1}X_{1}\gamma _{1}))\cdot \vdash _{\mathcal {M}}^{|\alpha _{1}|}((q,\omega _{1},\alpha _{1}X_{1}\gamma _{1}), (q,\omega _{2},X_{1}\gamma _{1}))\cdot \vdash _{\mathcal {M}} ((q,\omega _{2},X_{1}\gamma _{1}), (q,\omega _{2},\alpha _{2}X_{2}\gamma _{2}))\cdot \vdash _{\mathcal {M}}^{|\alpha _{2}|}((q,\omega _{3},\alpha _{2}X_{2}\gamma _{2}), (q,\omega _{3},X_{2}\gamma _{2}))\cdot \vdash _{\mathcal {M}}((q,\omega _{3},$$$$X_{2}\gamma _{2}),(q,\omega _{3}, \alpha _{3}X_{3}\gamma _{3}))\ldots \vdash _{\mathcal {M}}((q,\omega _{n},X_{n-1}\gamma _{n-1}),(q,\omega _{n},\alpha _{n}X_{n}\gamma _{n}))\cdot \vdash _{\mathcal {M}}^{|\alpha _{n}|}((q,\omega _{n},$$$$ \alpha _{n} X_{n}\gamma _{n}), (q,\omega _{n},X_{n}\gamma _{n}))\cdot \vdash _{\mathcal {M}}((q,\omega _{n},X_{n}\gamma _{n}), (q,\omega _{n},\omega _{n})) \cdot \vdash _{\mathcal {M}}^{|\omega _{n}|}((q,\omega _{n},\omega _{n}), (q,\varepsilon ,\varepsilon ))$$$$=k\in A{\setminus }\{0\}$$, where $$\omega =\alpha \omega _{1},\omega _{k-1}=\alpha _{k-1}\omega _{k},k=2,\ldots ,n$$, the mapping $$\vdash _{\mathcal {M}}^{|\alpha |}((q,\alpha ,\alpha X\gamma ), (q,\varepsilon ,$$$$X\gamma ))$$ is defined by,

$$\begin{aligned} \vdash _{\mathcal {M}}^{|\alpha |}((q,\alpha ,\alpha X\gamma ), (q,\varepsilon ,X\gamma ))=\left\{ \begin{array}{ll} 1, &{}\quad \hbox { if } |\alpha |=0,\\ h,&{}\quad \hbox { if } |\alpha |=k, \end{array}\right. \end{aligned}$$

where $$\alpha =a_{1}\ldots a_{k},a_{i}\in T, i=1,\ldots , k;$$ and $$h=\vdash _{\mathcal {M}}((q,\alpha , a_{1}\ldots a_{k} X\gamma ), (q,a_{2}\ldots a_{k},a_{2}\ldots a_{k}X\gamma ))\cdot \vdash _{\mathcal {M}}((q,a_{2}\ldots a_{k}, a_{2}\ldots a_{k} $$$$X\gamma ),(q,a_{3}\ldots a_{k},a_{3}\ldots a_{k}X\gamma ))\ldots \vdash _{\mathcal {M}}((q,a_{k},a_{k} X\gamma ), (q,\varepsilon ,X\gamma ))$$.

In fact, for a given $|\alpha |\ge 0,\ \alpha \in T^{*}, X\in N, \gamma \in \Gamma ^{*}$, there has $\vdash _{\mathcal {M}}^{|\alpha |}((q,\alpha ,\alpha X\gamma ), (q,\varepsilon ,X\gamma ))=1$.

For any $\omega \in T^{*}$, if $\rho (S\Rightarrow \omega )=k_{0}\in A{\setminus }\{0\}$, then $S\Rightarrow \omega$ if and only if $\vdash _{\mathcal {M}}((S,\omega ,Z_{0}),(q,\omega ,S))\cdot \vdash _{\mathcal {M}}((q,\omega ,S),(q,\omega ,\omega ))\cdot \vdash _{\mathcal {M}}^{|\omega |}((q,\omega ,\omega ),(q,\varepsilon ,\varepsilon ))=1\cdot \vdash _{\mathcal {M}}((q,\omega ,S),(q,\omega ,\omega ))\cdot 1=\vdash _{\mathcal {M}}((q,\varepsilon ,S),(q,\varepsilon ,\omega ))=\delta (q,\varepsilon , S,q,\omega ) =\rho (S\rightarrow \omega )=k_{0}.$

Hence, for any $$\omega \in T^{*}$$, there has $$\langle G\rangle _{r}^{L}(\omega )=\sum \{I(S)\cdot \rho (S\rightarrow \omega )|S\in N\}+\sum \{I(S)\cdot \rho (S\Rightarrow \alpha X\gamma \Rightarrow ^{L}\alpha \alpha _{1}X_{1}\gamma _{1}\Rightarrow ^{L} \alpha \alpha _{1}\alpha _{2}X_{2}\gamma _{2}\Rightarrow ^{L}\cdots \Rightarrow ^{L} \alpha \alpha _{1}\cdots \alpha _{n}X_{n}\gamma _{n}\Rightarrow ^{L}\omega )|S,X,X_{1},\ldots , X_{n}\in N, \alpha ,\alpha _{1},\ldots , \alpha _{n}\in T^{*},\gamma ,\gamma _{1},\ldots ,\gamma _{n}\in \Gamma ^{*}\} = \sum \{\sigma (S)\cdot \vdash _{\mathcal {M}}((S,\omega ,Z_{0}), (q,\omega ,S))\cdot \vdash _{\mathcal {M}}((q,\omega ,S),(q, \omega ,\omega ))\cdot \vdash _{\mathcal {M}}^{|\omega |}((q, \omega ,\omega ),(q,\varepsilon ,\varepsilon )) |S\in N\}+\sum \{\sigma (S)\cdot \vdash _{\mathcal {M}}((S,\omega ,Z_{0}),$$$$ (q,\omega ,S)) \cdot \vdash _{\mathcal {M}}((q,\omega ,S), (q,\omega ,\alpha X\gamma ))\cdot \vdash _{\mathcal {M}}^{|\alpha |}((q,\omega ,\alpha X\gamma ),(q,\omega _{1},X\gamma ))\cdot \vdash _{\mathcal {M}}((q,\omega _{1},$$$$ X\gamma ), (q,\omega _{1},\alpha _{1}X_{1}\gamma _{1}))\cdot \vdash _{\mathcal {M}}^{|\alpha _{1}|}((q,\omega _{1},\alpha _{1}X_{1}\gamma _{1}), (q,\omega _{2},X_{1}\gamma _{1})) \cdot \vdash _{\mathcal {M}}((q,\omega _{2},X_{1}\gamma _{1}),$$$$ (q,\omega _{2},\alpha _{2}X_{2}\gamma _{2})) \cdot \vdash _{\mathcal {M}}^{|\alpha _{2}|}((q,\omega _{3},\alpha _{2}X_{2}\gamma _{2}), (q,\omega _{3},X_{2}\gamma _{2}))\cdot \vdash _{\mathcal {M}}((q,\omega _{3},X_{2} \gamma _{2}),(q,\omega _{3},$$$$\alpha _{3}X_{3}\gamma _{3})) \ldots \vdash _{\mathcal {M}}((q,\omega _{n},X_{n-1}\gamma _{n-1}), (q,\omega _{n},\alpha _{n}X_{n}\gamma _{n})) \cdot \vdash _{\mathcal {M}}^{|\alpha _{n}|}((q,\omega _{n},\alpha _{n}X_{n}\gamma _{n}),$$$$ (q,\omega _{n}, X_{n}\gamma _{n}))\cdot \vdash _{\mathcal {M}} ((q,\omega _{n},X_{n}\gamma _{n}), (q,\omega _{n},\omega _{n}))\cdot \vdash _{\mathcal {M}}^{|\omega _{n}|}((q, \omega _{n},\omega _{n}), (q,\varepsilon ,\varepsilon )) |S,X,$$$$X_{1},\ldots , X_{n}\in N, \alpha ,\alpha _{1},\ldots , \alpha _{n}\in T^{*},\gamma ,\gamma _{1},\ldots ,\gamma _{n}\in \Gamma ^{*}, \omega =\alpha \omega _{1},\omega _{k-1}=\alpha _{k-1}\omega _{k},k=2,\ldots ,n \}=\langle rec(\mathcal {M})\rangle _{r}(\omega )$$.

So $$\langle rec(\mathcal {M})\rangle _{r}=\langle G\rangle _{r}^{L}$$.

Similarly it follows by definition that $\langle rec(\mathcal {M})\rangle _{b}=\langle G\rangle _{b}^{L}$.

### **Proposition 9**

*Let*$\mathcal {M}$*be a*$\hbox {WPDA}^{\emptyset }$*over a complete strong bimonoidA*. *Then there exists a WCFG*$G=(N,T,P,I)$*such that*$\langle rec(\mathcal {M})\rangle _{x}=\langle G\rangle _{x}^{L},\ \forall x\in \{r,b\}$.

### *Proof*

Let $\mathcal {M}=(Q,\Sigma ,\Gamma ,\delta ,\sigma ,Z_{0},\emptyset )$ be a WPDA$^{\emptyset }$ over a complete strong bimonoid *A*. Then a WCFG $G=(N,T,P,I)$ over a complete strong bimonoid *A* is established as follows: $N=Q\cup \{[pzq]|p,q\in Q,z\in \Gamma \}$, $T=\Sigma$,

$$\begin{aligned} I(x)=\left\{ \begin{array}{ll} \sigma (x), &{}\quad \hbox { if } x\in Q,\\ 0,&{} \quad \hbox { if } x\in N{\setminus } Q \end{array}\right. \end{aligned}$$

*P* consists of the following productions:

(i) $$p\overset{1}{\rightarrow }[pZ_{0}q]$$, $$\forall p,q\in Q$$;
(ii) for any $$p,q\in Q, u\in \Sigma \cup \{\varepsilon \}$$, $$Z,Z_{1},\ldots ,Z_{m}\in \Gamma $$, $$m\ge 1$$, if $$\delta (p,u,Z,q,Z_{1}\ldots Z_{m})$$$$=l\in A{\setminus }\{0\}$$, then $$[pZq_{m}]\overset{l}{\rightarrow }u[qZ_{1}q_{1}][q_{1}Z_{2}q_{2}] \cdots [q_{m-1}Z_{m}q_{m}], \forall q_{1},\ldots , q_{m}\in Q.$$
(iii) if $\delta (p,u,Z,q,\varepsilon )=l\in A{\setminus }\{0\}$, then $[pZq]\overset{l}{\rightarrow }u$.

For $$\omega =u_{1}u_{2}\ldots u_{m}$$, $$m\ge 2$$, $$u_{i}\in \Sigma \cup \{\varepsilon \}$$, $$i=1,\ldots ,m$$, assume the set $$^{m}Path_{\omega }^{l}(pZq)$$ consists of all the traces that inputting string $$\omega \in \Sigma ^{*}$$ makes state *p* transfer to state *q* and pushes the top symbol *Z* out of the stack, where the string $$\omega $$ is a sequence catenated by m elements from $$\Sigma \cup \{\varepsilon \}$$, and the truth value of all the above propositions are *l*. That is to say, $$^{m}Path_{\omega }^{l}(pZq)=\{(p_{1},u_{2}\ldots u_{m},Z_{1}\gamma _{1},p_{2},u_{3}\ldots u_{m},Z_{2}\gamma _{2}, \ldots ,p_{m-2},u_{m-1}u_{m}, Z_{m-2}$$$$\gamma _{m-2}, p_{m-1},u_{m},Z_{m-1},q,\varepsilon ,\varepsilon )| \vdash _{\mathcal {M}}((p,\omega ,Z),(p_{1},u_{2}\ldots u_{m},Z_{1}\gamma _{1}))\cdot \vdash _{\mathcal {M}}((p_{1},u_{2}$$$$\ldots u_{m}, Z_{1}\gamma _{1}), (p_{2},u_{3}\ldots u_{m},Z_{2}\gamma _{2}))\ldots \vdash _{\mathcal {M}}((p_{m-2},u_{m-1}u_{m}, Z_{m-2}\gamma _{m-2}), (p_{m-1},u_{m},$$$$Z_{m-1})) \cdot \vdash _{\mathcal {M}}((p_{m-1},u_{m},Z_{m-1}), (q,\varepsilon ,\varepsilon ))=l\in A{\setminus }\{0\}, p_{1},\ldots ,p_{m-1}\in Q, Z_{1},\ldots ,$$$$Z_{m-1}\in \Gamma ,\gamma _{1},\gamma _{2}, \ldots ,\gamma _{m-2}\in \Gamma ^{*}\}$$.

Then for any $$t(\omega )\!\in \!\ ^{m}Path_{\omega }^{l}(pZq)$$, we define $$\vdash _{\mathcal {M}}^{t(\omega )}((p,\omega ,Z),(q,\varepsilon ,\varepsilon )) =\vdash _{\mathcal {M}}((p,\omega ,Z),(p_{1},u_{2}\ldots u_{m}, Z_{1}\gamma _{1}))\cdot \vdash _{\mathcal {M}}((p_{1},u_{2}\ldots u_{m},$$$$ Z_{1}\gamma _{1}), (p_{2},u_{3}\ldots u_{m}, Z_{2}\gamma _{2}))\ldots \vdash _{\mathcal {M}}((p_{m-2}, u_{m-1}u_{m},Z_{m-2}\gamma _{m-2}), (p_{m-1}, u_{m},Z_{m-1}))$$$$ \cdot \vdash _{\mathcal {M}}((p_{m-1},u_{m},Z_{m-1}),(q,\varepsilon ,\varepsilon ))$$.

If $t(\omega )\in \{(q,\varepsilon ,\varepsilon )|\vdash _{\mathcal {M}}((p,\omega ,Z),(q,\varepsilon ,\varepsilon ))=l\in A{\setminus }\{0\}\}$, then we define $\vdash _{\mathcal {M}}^{t(\omega )}((p,\omega ,Z),(q,\varepsilon ,\varepsilon )) =\vdash _{\mathcal {M}}((p,\omega ,Z),(q,\varepsilon ,\varepsilon ))$.

Assume that $$^{m}Dr_{\omega }^{l}(pZq)=\{(u_{1}[p_{1}Z_{1}p_{1}^{'}]\alpha _{1}, u_{2}[p_{2}Z_{2}p_{2}^{'}]\alpha _{2},\ldots , u_{m-2}[p_{m-2}Z_{m-2}p_{m-2}^{'}]\alpha _{m-2}, u_{m-1}$$$$\alpha _{m-1}, u_{m} )|\rho ([pZq] \Rightarrow ^{L}u_{1}[p_{1}Z_{1}p_{1}^{'}]\alpha _{1}\Rightarrow ^{L} u_{1}u_{2}[p_{2}Z_{2}p_{2}^{'}]\alpha _{2}\Rightarrow ^{L}\cdots \Rightarrow ^{L} u_{1}u_{2}\ldots u_{m-2}$$$$[p_{m-2}Z_{m-2}p_{m-2}^{'}]\alpha _{m-2} \Rightarrow ^{L} u_{1}u_{2}\ldots u_{m-2} u_{m-1}\alpha _{m-1}\Rightarrow ^{L} u_{1}u_{2}\ldots u_{m-2}u_{m-1}u_{m})=l\in A{\setminus }\{0\}, \alpha _{1},\alpha _{2},\ldots ,\alpha _{m-2}\in N^{*}, \alpha _{m-1}=[p_{m-1}Z_{m-1}q], p_{1},p_{2}, \ldots ,p_{m-1}\in Q, p_{1}^{'},p_{2}^{'}, \ldots , p_{m-2}^{'}\in Q\}$$.

For any $$c(\omega )\in \ ^{m}Dr_{\omega }^{l}(pZq)$$, define $$\rho ([pZq]\Rightarrow _{c(\omega )}^{*L}\omega )=\rho ([pZq]\Rightarrow ^{L}u_{1}[p_{1}Z_{1}p_{1}^{'}]\alpha _{1}\Rightarrow ^{L} u_{1}u_{2}[p_{2}Z_{2}p_{2}^{'}]\alpha _{2}\Rightarrow ^{L}\ldots \Rightarrow ^{L} u_{1}u_{2}\ldots u_{m-2}[p_{m-2}Z_{m-2}p_{m-2}^{'}]\alpha _{m-2}\Rightarrow ^{L} u_{1}u_{2}\ldots u_{m-2}u_{m-1}\alpha _{m-1}\Rightarrow ^{L} u_{1}u_{2}\ldots u_{m-2}$$$$u_{m-1}u_{m})$$.

If $c(\omega )\in \{\omega |\rho ([pZq]\Rightarrow \omega )=l\in A{\setminus }\{0\}\}$, then we define $\rho ([pZq]\Rightarrow _{c(\omega )}^{*L}\omega )=\rho ([pZq]\Rightarrow \omega )$.

Now the following statements hold:

(I) $\forall \omega \in \Sigma ^{*}, p,q\in Q$, $\vdash _{\mathcal {M}}((p,\omega ,Z_{0}),(q,\varepsilon ,\varepsilon ))=l\in A{\setminus }\{0\}$ if and only if $\rho ([pZ_{0}q]\Rightarrow ^{L}\omega )=l\in A{\setminus }\{0\}$.

(II) Put $$Path_{\omega }^{l}(pZq)=\bigcup \{^{m}Path_{\omega }^{l}(pZq)|m\ge 2\}$$, and $$Dr_{\omega }^{l}(pZq)=\bigcup \{^{m}Dr_{\omega }^{l}(pZq)$$$$|m\ge 2\}$$. Then for any $$\omega =u_{1}u_{2}\ldots u_{k}\in \Sigma ^{*}$$, $$p,q\in Q$$, $$z\in \Gamma $$, $$l\in A{\setminus }\{0\}$$, $$u_{i}\in \Sigma \cup \{\varepsilon \}$$, $$i=1,\ldots ,k, k\ge 2$$, there exists a bijective mapping *f* from $$Path_{\omega }^{l}(pZq)$$ to $$Dr_{\omega }^{l}(pZq)$$, i.e., $$f:Path_{\omega }^{l}(pZq)\rightarrow Dr_{\omega }^{l}(pZq)$$, which is given by $$f((p_{1},u_{2}\ldots u_{m},Z_{1}\gamma _{1},p_{2},u_{3}\ldots u_{m},Z_{2}\gamma _{2},\ldots ,p_{m-2}, u_{m-1}u_{m}, Z_{m-2}\gamma _{m-2}, p_{m-1}, u_{m},$$$$Z_{m-1},q,\varepsilon ,\varepsilon ))\!=\! (u_{1}[p_{1}Z_{1}p_{1}^{'}]\alpha _{1}, u_{2}[p_{2}Z_{2}p_{2}^{'}]\alpha _{2},\ldots , u_{m-2}[p_{m-2}Z_{m-2}p_{m-2}^{'}] \alpha _{m-2}, u_{m-1}$$$$\alpha _{m-1},u_{m})$$, $$\forall (p_{1},u_{2}\ldots u_{m},Z_{1}\gamma _{1},p_{2}, u_{3}\ldots u_{m},Z_{2}\gamma _{2},\ldots ,p_{m-2}, u_{m-1}u_{m}, Z_{m-2}\gamma _{m-2}, p_{m-1},$$$$ u_{m},Z_{m-1},q,\varepsilon ,\varepsilon )\in Path_{\omega }^{l}(pZq)$$, where $$p_{1}^{'},p_{2}^{'},\ldots ,p_{m-2}^{'},\alpha _{1},\alpha _{2},\ldots ,\alpha _{m-2}$$ can be determined uniquely by the productions in *P* respectively.

Clearly, for any $$(p_{1},u_{2}\ldots u_{m},Z_{1}\gamma _{1},p_{2},u_{3}\ldots u_{m}, Z_{2}\gamma _{2},\ldots ,p_{m-2}, u_{m-1}u_{m}, Z_{m-2}\gamma _{m-2},$$$$ p_{m-1},u_{m},Z_{m-1}, q,\varepsilon ,\varepsilon )\in \ ^{m}Path_{\omega }^{l}(pZq)$$, there always has $$f((p_{1},u_{2}\ldots u_{m},Z_{1}\gamma _{1},p_{2},u_{3}\ldots u_{m}, Z_{2}\gamma _{2},\ldots ,p_{m-2}, u_{m-1}u_{m}, Z_{m-2}$$$$\gamma _{m-2}, p_{m-1},u_{m}, Z_{m-1}, q,\varepsilon ,\varepsilon ))\in \ ^{m}Dr_{\omega }^{l}(pZq)$$.

Next *f* is shown to be bijective by induction. In fact, if $Path_{\omega }^{l}(pZq)=\emptyset$, then $Dr_{\omega }^{l}(pZq)=\emptyset$; if $Path_{\omega }^{l}(pZq)\ne \emptyset$, then $Dr_{\omega }^{l}(pZq)\ne \emptyset$.

Basis: if $$t(\omega )=(p_{1},u_{2},Z_{1},q,\varepsilon ,\varepsilon )\in \ ^{2}Path_{\omega }^{l}(pZq)$$, then $$\vdash _{\mathcal {M}}^{t(\omega )}((p,\omega ,Z),(q,\varepsilon ,\varepsilon )) $$$$=\vdash _{\mathcal {M}}((p,\omega ,Z),(p_{1},u_{2},Z_{1})) \cdot \vdash _{\mathcal {M}}((p_{1},u_{2},Z_{1}),(q,\varepsilon ,\varepsilon ))=l$$, and so $$[pZp^{'}]\rightarrow u_{1}[p_{1}Z_{1}p^{'}]$$, $$[p_{1}Z_{1}q]\rightarrow u_{2}$$, and $$\rho ([pZp^{'}]\rightarrow u_{1}[p_{1}Z_{1}p^{'}])=\vdash _{\mathcal {M}}((p,\omega ,Z),(p_{1},u_{2},Z_{1}))$$, $$\rho ([p_{1}Z_{1}q]\rightarrow u_{2})=\vdash _{\mathcal {M}}((p_{1},u_{2},Z_{1}),(q,\varepsilon ,\varepsilon ))$$.

Suppose $\alpha _{1}=[p_{1}Z_{1}q]$, and $p^{'}=q$. Then $c(\omega )=(u_{1}\alpha _{1},u_{2})=f(t(\omega ))$, $\rho ([pZq]\Rightarrow _{c(\omega )}^{*L}\omega )=\rho ([pZq]\Rightarrow ^{L}u_{1}\alpha _{1}\Rightarrow ^{L}u_{1}u_{2})=l$.

Clearly, for any $$x,y\in $$$$^{2}Path_{\omega }^{l}(pZq)$$, if $$x\ne y$$, then $$f(x)\ne f(y)$$. Moreover, for any $$y\in \ ^{2}Dr_{\omega }^{l}(pZq)$$, there exists $$x\in \ ^{2}Path_{\omega }^{l}(pZq)$$ such that $$f(x)=y$$. So the mapping $$f\mid _{^{2}Path_{\omega }^{l}(pZq)}$$, named as a restriction on a set $$^{2}Path_{\omega }^{l}(pZq)$$ by *f*, is a bijective mapping from $$^{2}Path_{\omega }^{l}(pZq)$$ to $$^{2}Dr_{\omega }^{l}(pZq)$$.

Assumption by induction: Assume that whenever the derivation step number from $$(p,\omega ,Z)$$ to $$(q,\varepsilon ,\varepsilon )$$ is not more than *k* ($$k\ge 2$$) in the given WPDA$$^{\emptyset }$$$$\mathcal {M}$$, there exists a bijective map $$f\mid _{^{m}Path_{\omega }^{l}(pZq)}:\ ^{m}Path_{\omega }^{l}(pZq)\rightarrow \ ^{m}Dr_{\omega }^{l}(pZq)$$, $$m=2,\ldots ,k$$. Next we prove $$f\mid _{^{k+1}Path_{\omega }^{l}(pZq)}:\ ^{k+1}Path_{\omega }^{l}(pZq)\rightarrow \ ^{k+1}Dr_{\omega }^{l}(pZq)$$ is also bijective, named as a restriction on the set $$^{k+1}Path_{\omega }^{l}(pZq)$$ by *f*.

In fact, if $$t(\omega )\in \ ^{k+1}Path_{\omega }^{l}(pZq)$$, then $$\vdash _{\mathcal {M}}^{t(\omega )}((p,\omega ,Z),(q,\varepsilon ,\varepsilon ))= \vdash _{\mathcal {M}}((p,a\tau _{1}\tau _{2}\ldots $$$$\tau _{m},Z),(q_{1}, \tau _{1}\tau _{2}\ldots \tau _{m}, X_{1}X_{2}\ldots X_{m}))\cdot \vdash _{\mathcal {M}}^{t(\tau _{1})}((q_{1},\tau _{1}\tau _{2}\ldots \tau _{m}, X_{1}X_{2} \ldots X_{m}),(q_{2},\tau _{2}\ldots $$$$\tau _{m}, X_{2}\ldots X_{m}))\cdot \vdash _{\mathcal {M}}^{t(\tau _{2})}((q_{2},\tau _{2}\ldots \tau _{m}, X_{2}\ldots X_{m}), (q_{3},\tau _{3}\ldots \tau _{m}, X_{3}\ldots X_{m}))\ldots \vdash _{\mathcal {M}}^{t(\tau _{m})}((q_{m}, \tau _{m}, X_{m}),(q,\varepsilon , \varepsilon ))=l$$, where $$a\in \Sigma \cup \{\varepsilon \}$$, $$\tau _{1},\ldots ,\tau _{m}\in \Sigma ^{*}$$, $$X_{1},X_{2},\ldots ,X_{m}\in \Gamma $$, $$q_{1},\ldots ,q_{m}\in Q$$, $$\omega =a\tau _{1}\ldots \tau _{m}$$.

Let $$\vdash _{\mathcal {M}}((p,a,Z),(q_{1},\varepsilon , X_{1}X_{2}\ldots X_{m}))=l_{a}$$, $$\vdash _{\mathcal {M}}^{t(\tau _{i})}((q_{i},\tau _{i},X_{i}),(q_{i+1},\varepsilon , \varepsilon ))=l_{i}$$, $$t(\tau _{i})\in \ ^{k_{i}}Path_{\tau _{i}}^{l_{i}}(q_{i}X_{i}q_{i+1})\cup D_{i}$$, $$D_{i}=\{(q_{i+1},\varepsilon ,\varepsilon )|\vdash _{\mathcal {M}}((q_{i},\tau _{i}, X_{i}),(q_{i+1},\varepsilon , \varepsilon ))=l_{i}\in A{\setminus }\{0\}\}$$, $$i=1,\ldots ,m,$$$$q_{m+1}=q$$. Then $$l_{a}\cdot l_{1}\ldots l_{m}=l$$, $$t(\omega )\in \ ^{k+1}Path_{\omega }^{l}(pZq)=H\times A_{1}\times A_{2}\times \cdots \times A_{m}$$, where $$H=\{(q_{1},\tau _{1}\ldots \tau _{m}, X_{1}X_{2}\ldots X_{m})|\vdash _{\mathcal {M}}((p,a\tau _{1}\tau _{2}\ldots \tau _{m}, Z),(q_{1},\tau _{1}\tau _{2}\ldots \tau _{m}, X_{1}X_{2}$$$$\ldots X_{m}))=l_{a}\in A{\setminus }\{0\}, \tau _{1},\ldots , \tau _{m}\in \Sigma ^{*},X_{1},X_{2},\ldots , X_{m}\in \Gamma ,a\tau _{1}\tau _{2}\ldots \tau _{m}=\omega ,a\in \Sigma \cup \{\varepsilon \}, q_{1}\in Q\}$$;

$$A_{1}=\{(p_{11},u_{12}\ldots u_{1k_{1}}\tau _{2}\ldots \tau _{m},Z_{11}\gamma _{11}X_{2}\ldots X_{m},p_{12}, u_{13}\ldots u_{1k_{1}}\tau _{2}\ldots \tau _{m}, Z_{12}\gamma _{12}X_{2}$$$$\ldots X_{m},\ldots ,p_{1,k_{1}-2}, u_{1,k_{1}-1} u_{1,k_{1}}\tau _{2}\ldots \tau _{m}, Z_{1,k_{1}-2}\gamma _{1,k_{1}-2}X_{2}\ldots X_{m}, p_{1,k_{1}-1}, u_{1,k_{1}}\tau _{2}\ldots \tau _{m},$$$$Z_{1,k_{1}-1}X_{2}\ldots X_{m},q_{2},\tau _{2}\ldots \tau _{m},X_{2}\ldots X_{m})| t(\tau _{1})\!=\!(p_{11}, u_{12}\ldots u_{1k_{1}}, Z_{11}\gamma _{11},p_{12}, u_{13}\ldots $$$$u_{1k_{1}},Z_{12}\gamma _{12}, \ldots ,p_{1,k_{1}-2}, u_{1,k_{1}-1}u_{1,k_{1}}, Z_{1,k_{1}-2}\gamma _{1,k_{1}-2}, p_{1,k_{1}-1},u_{1,k_{1}}, Z_{1,k_{1}-1},q_{2},\varepsilon ,\varepsilon )\in \ ^{k_{1}}Path_{\tau _{1}}^{l_{1}}(q_{1}X_{1}q_{2}), \tau _{1}=u_{11}u_{12}\ldots u_{1k_{1}}\}\cup \{(q_{2},\tau _{2}\ldots \tau _{m},X_{2}\ldots X_{m})|t(\tau _{1})\in D_{1}\}$$;

$$A_{2}=\{(p_{21},u_{22}\ldots u_{2k_{2}}\tau _{3}\ldots \tau _{m},Z_{21}\gamma _{21}X_{3}\ldots X_{m},p_{22}, u_{23}\ldots u_{2k_{2}}\tau _{3}\ldots \tau _{m}, Z_{22}\gamma _{22}$$$$X_{3}\ldots X_{m},\ldots , p_{2,k_{2}-2}, u_{2,k_{2}-1} u_{2,k_{2}}\tau _{3}\ldots \tau _{m}, Z_{2,k_{2}-2}\gamma _{2,k_{2}-2}X_{3}\ldots X_{m}, p_{2,k_{2}-1}, u_{2,k_{2}}\tau _{3}\ldots $$$$\tau _{m},Z_{2,k_{2}-1}X_{3}\ldots X_{m},q_{3},\tau _{3}\ldots \tau _{m}, X_{3}\ldots X_{m})|t(\tau _{2})\!=\! (p_{21}, u_{22}\ldots u_{2k_{2}}, Z_{21}\gamma _{21},p_{22}, u_{23}$$$$\ldots u_{2k_{2}},Z_{22}\gamma _{22},\ldots , p_{2,k_{2}-2}, u_{2,k_{2}-1}u_{2,k_{2}}, Z_{2,k_{2}-2}\gamma _{2,k_{2}-2}, p_{2,k_{2}-1},u_{2,k_{2}}, Z_{2,k_{2}-1},q_{3},\varepsilon ,\varepsilon )$$$$\in ^{k_{2}}Path_{\tau _{2}}^{l_{2}}(q_{2}X_{2}q_{3}), \tau _{2}=u_{21}u_{22}\ldots u_{2k_{2}}\}\cup \{(q_{3},\tau _{3}\ldots \tau _{m},X_{3}\ldots X_{m})|t(\tau _{2})\in D_{2}\}$$;

$$\vdots $$

$$A_{m-1}=\{(p_{m-1,1},u_{m-1,2}\ldots u_{m-1,k_{m-1}}\tau _{m},Z_{m-1,1} \gamma _{m-1,1} X_{m},p_{m-1,2}, u_{m-1,3}\ldots u_{m-1,k_{m-1}}$$$$\tau _{m}, Z_{m-1,2} \gamma _{m-1,2} X_{m},\ldots , p_{m-1,k_{m-1}-2},u_{m-1,k_{m-1}-1}u_{m-1,k_{m-1}}\tau _{m}, Z_{m-1,k_{m-1}-2} \gamma _{m-1,k_{m-1}-2}$$$$ X_{m}, p_{m-1,k_{m-1}-1}, u_{m-1,k_{m-1}} \tau _{m},Z_{m-1,k_{m-1}-1} X_{m},q_{m},\tau _{m},X_{m})| t(\tau _{m-1})=(p_{m-1,1}, u_{m-1,2}$$$$\ldots u_{m-1,k_{m-1}},\ Z_{m-1,1}\gamma _{m-1,1},\ p_{m-1,2},\ u_{m-1,3}\ldots u_{m-1,k_{m-1}},\ Z_{m-1,2}\gamma _{m-1,2},\ \ldots ,p_{m-1,k_{m-1}-2}, $$$$u_{m-1,k_{m-1}-1} u_{m-1,k_{m-1}}, Z_{m-1,k_{m-1}-2}\gamma _{m-1,k_{m-1}-2}, p_{m-1,k_{m-1}-1}, u_{m-1,k_{m-1}}, Z_{m-1,k_{m-1}-1}, q_{m},$$$$\varepsilon ,\varepsilon )\in \ ^{k_{m-1}}Path_{\tau _{m-1}}^{l_{m-1}} (q_{m-1}X_{m-1}q_{m}), \tau _{m-1}=u_{m-1,1}u_{m-1,2}\ldots u_{m-1,k_{m-1}}\}\cup \{(q_{m},\tau _{m}, X_{m})$$$$|t(\tau _{m-1})\in D_{m-1}\}$$;

$$A_{m}=\{(p_{m1},u_{m2}\ldots u_{mk_{m}},Z_{m1}\gamma _{m1},p_{m2},u_{m3}\ldots u_{mk_{m}}, Z_{m2}\gamma _{m2},\ldots ,p_{m,k_{m}-2}, u_{m,k_{m}-1}$$$$u_{m,k_{m}}, Z_{m,k_{m}-2} \gamma _{m,k_{m}-2}, p_{m,k_{m}-1}, u_{m,k_{m}},Z_{m,k_{m}-1},q,\varepsilon ,\varepsilon )|t(\tau _{m})= (p_{m1},u_{m2}\ldots u_{mk_{m}}, $$$$Z_{m1}\gamma _{m1},p_{m2}, u_{m3}\ldots u_{mk_{m}},\ Z_{m2}\gamma _{m2},\ \ldots ,\ p_{m,k_{m}-2},\ u_{m,k_{m}-1}u_{m,k_{m}},\ Z_{m,k_{m}-2}\gamma _{m,k_{m}-2},\ p_{m,k_{m}-1},\ $$$$u_{m,k_{m}},Z_{m,k_{m}-1},q,\varepsilon ,\varepsilon )\in \ ^{k_{m}}Path_{\tau _{m}}^{l_{m}}(q_{m}X_{m}q), \tau _{m}=u_{m1}u_{m2}\ldots u_{mk_{m}}\}\cup \{(q,\varepsilon ,\varepsilon )|t(\tau _{m})\in D_{m}\}$$.

Let $$F_{i}=\{\tau _{i}|\rho ([q_{i}X_{i}q_{i+1}]\Rightarrow \tau _{i})=l_{i}\in A{\setminus }\{0\}\}, i=1,\ldots ,m,$$ and $$q_{m+1}=q$$.

Then it is obtained by induction that, $$\rho \left( [q_{1}X_{1}q_{2}]\Rightarrow _{c(\tau _{1})}^{*L}\tau _{1}\right) \!=\!l_{1}$$, $$\rho \left( [q_{2}X_{2}q_{3}]\Rightarrow _{c(\tau _{2})}^{*L}\tau _{2}\right) =l_{2},\ldots $$, $$\rho \left( [q_{m}X_{m}q]\Rightarrow _{c(\tau _{m})}^{*L}\tau _{m}\right) $$$$=l_{m}$$, $$c(\tau _{1})\in \ ^{k_{1}}Dr_{\tau _{1}}^{l_{1}}(q_{1}X_{1}q_{2})\cup F_{1}$$, $$c(\tau _{2})\in \ ^{k_{2}}Dr_{\tau _{2}}^{l_{2}}(q_{2}X_{2}q_{3})\cup F_{2},\ldots $$, $$c(\tau _{m})\in \ ^{k_{m}}Dr_{\tau _{m}}^{l_{m}}(q_{m}X_{m}q)\cup F_{m}$$.

So $$\rho \left( [pZq]\Rightarrow _{c(\omega )}^{*L}\omega \right) =\rho \left( [pZq]\Rightarrow ^{L}a[q_{1}X_{1}q_{2}][q_{2}X_{2}q_{3}] \ldots [q_{m} X_{m}q]\Rightarrow _{c(\tau _{1})}^{*L}a\tau _{1}[q_{2}X_{2}\right. q_{3}]$$$$\ldots [q_{m}X_{m}q] \Rightarrow _{c(\tau _{2})}^{*L} a\tau _{1}\tau _{2}[q_{3}X_{3}q_{4}]\ldots [q_{m}X_{m}q]\Rightarrow _{c(\tau _{3})}^{*L}\cdots \Rightarrow _{c(\tau _{m-1})}^{*L} a\tau _{1}\ldots \tau _{m-1} [q_{m}X_{m}q]$$$$\left. \Rightarrow _{c(\tau _{m})}^{*L}a\tau _{1}\ldots \tau _{m}\right) =l_{a}\cdot l_{1}\ldots l_{m}=l$$, where $$c(\omega )\in \ ^{k+1}Dr_{\omega }^{l}(pZq)= E\times B_{1}\times B_{2}\times \cdots \times B_{m}$$, $$E\!=\!\{a[q_{1}X_{1}q_{2}][q_{2}X_{2}q_{3}]\ldots [q_{m-1}X_{m-1}q_{m}] [q_{m}X_{m}q] |\rho ([pZq]\Rightarrow ^{L}a[q_{1}X_{1}q_{2}][q_{2}X_{2}$$$$q_{3}] \ldots [q_{m-1}X_{m-1}q_{m}][q_{m} X_{m}q])=l_{a},a\in \Sigma \cup \{\varepsilon \}, q_{1},q_{2},\ldots ,q_{m}\in Q, X_{1},\ldots , X_{m}\in \Gamma \}$$, $$B_{1}\!=\!\{(u_{11}[p_{11}Z_{11}p_{11}^{'}]\alpha _{11}[q_{2}X_{2}q_{3}]\ldots [q_{m-1}X_{m-1}q_{m} ] [q_{m}X_{m}q], u_{12}[p_{12}Z_{12}p_{12}^{'}] \alpha _{12}[q_{2}$$$$X_{2}q_{3}]\ldots [q_{m-1}X_{m-1}q_{m}] [q_{m}X_{m}q],\ldots , u_{1,k_{1}-2}[p_{1,k_{1}-2}Z_{1,k_{1}-2}p_{1,k_{1}-2}^{'}]\alpha _{1,k_{1}-2}[q_{2} X_{2}q_{3}]\ldots $$$$[q_{m-1}X_{m-1}q_{m}][q_{m}X_{m}q], u_{1,k_{1}-1}\alpha _{1,k_{1}-1}[q_{2}X_{2} q_{3}]\ldots [q_{m-1}X_{m-1}q_{m}][q_{m}X_{m}q], u_{1k_{1}}[q_{2}X_{2}q_{3}]$$$$\ldots [q_{m-1} X_{m-1}q_{m}][q_{m}X_{m}q]) |c(\tau _{1})=(u_{11}[p_{11}Z_{11}p_{11}^{'}]\alpha _{11}, u_{12}[p_{12} Z_{12}p_{12}^{'}]\alpha _{12},\ldots , u_{1,k_{1}-2}$$$$[p_{1,k_{1}-2}Z_{1,k_{1}-2}p_{1,k_{1}-2}^{'}]\alpha _{1,k_{1}-2}, u_{1,k_{1}-1}\alpha _{1,k_{1}-1}, u_{1k_{1}})\in \ ^{k_{1}}Dr_{\tau _{1}}^{l_{1}}(q_{1}X_{1}q_{2})\}\cup \{\tau _{1}[q_{2}X_{2}q_{3}] \ldots $$$$[q_{m-1}X_{m-1}q_{m}][q_{m}X_{m}q]|c(\tau _{1})\in F_{1}\}$$,

$$B_{2}\!=\!\{(u_{21}[p_{21}Z_{21}p_{21}^{'}]\alpha _{21}[q_{3}X_{3}q_{4}]\ldots [q_{m-1}X_{m-1}q_{m} ][q_{m}X_{m}q], u_{22}[p_{22}Z_{22}p_{22}^{'}] \alpha _{22}$$$$[q_{3}X_{3}q_{4}]\ldots [q_{m-1}X_{m-1}q_{m}] [q_{m}X_{m}q],\ldots , u_{2,k_{2}-2}[p_{2,k_{2}-2}Z_{2,k_{2}-2}p_{2,k_{2}-2}^{'}]\alpha _{2,k_{2}-2} [q_{3} X_{3}q_{4}]$$$$\ldots [q_{m-1}X_{m-1}q_{m}][q_{m}X_{m}q], u_{2,k_{2}-1}\alpha _{2,k_{2}-1}[q_{3}X_{3} q_{4}]\ldots [q_{m-1}X_{m-1}q_{m}][q_{m}X_{m}q],u_{2k_{2}}[q_{3}$$$$X_{3}q_{4}]\ldots [q_{m-1} X_{m-1}q_{m}][q_{m}X_{m}q] ) |c(\tau _{2})=(u_{21}[p_{21}Z_{21}p_{21}^{'}]\alpha _{21}, u_{22}[p_{22} Z_{22}p_{22}^{'}]\alpha _{22},\ldots ,$$$$ u_{2,k_{2}-2}[p_{2,k_{2}-2}Z_{2,k_{2}-2}p_{2,k_{2}-2}^{'}]\alpha _{2,k_{2}-2}, u_{2,k_{2}-1}\alpha _{2,k_{2}-1}, u_{2k_{2}})\in \ ^{k_{2}}Dr_{\tau _{2}}^{l_{2}}(q_{2}X_{2}q_{3})\}\cup \{\tau _{2}[q_{3}$$$$X_{3}q_{4}] \ldots [q_{m-1}X_{m-1}q_{m}][q_{m}X_{m}q]|c(\tau _{2})\in F_{2}\}$$,

$$\vdots $$

$$B_{m-1}=\{(u_{m-1,1}[p_{m-1,1}Z_{m-1,1}p_{m-1,1}^{'}] \alpha _{m-1,1}[q_{m} X_{m}q], u_{m-1,2}[p_{m-1,2}Z_{m-1,2}p_{m-1,2}^{'}]$$$$\alpha _{m-1,2} [q_{m}X_{m}q],\ldots , u_{m-1,k_{m-1}-2}[p_{m-1,k_{m-1}-2}Z_{m-1,k_{m-1}-2}p_{m-1,k_{m-1}-2}^{'}] \alpha _{m-1,k_{m-1}-2}[q_{m}$$$$X_{m}q], u_{m-1,k_{m-1}-1}\alpha _{m-1,k_{m-1}-1}[q_{m} X_{m}q], u_{m-1,k_{m-1}}[q_{m}X_{m}q] ) |c(\tau _{m-1})=(u_{m-1,1}[p_{m-1,1} $$$$Z_{m-1,1}p_{m-1,1}^{'}]\alpha _{m-1,1}, u_{m-1,2}[p_{m-1,2}Z_{m-1,2}p_{m-1,2}^{'}] \alpha _{m-1,2}, \ldots , u_{m-1,k_{m-1}-2}[p_{m-1,k_{m-1}-2}$$$$Z_{m-1,k_{m-1}-2} p_{m-1,k_{m-1}-2}^{'}] \alpha _{m-1,k_{m-1}-2}, u_{m-1,k_{m-1}-1}\alpha _{m-1,k_{m-1}-1}, u_{m-1,k_{m-1}})\in \ ^{k_{m-1}}Dr_{\tau _{m-1}}^{l_{m-1}}$$$$(q_{m-1}X_{m-1}q_{m})\}\cup \{ \tau _{m-1}[q_{m} X_{m}q]|c(\tau _{m-1})\in F_{m-1}\}$$, $$B_{m}=\ ^{k_{m}}Dr_{\tau _{m}}^{l_{m}}(q_{m}X_{m}q)\cup \{\tau _{m}|c(\tau _{m})\in F_{m}\}$$.

Since $$f\mid _{^{k_{i}}Path_{\tau _{i}}^{l_{i}}(q_{i}X_{i}q_{i+1})}$$ is a bijective mapping from $$^{k_{i}}Path_{\tau _{i}}^{l_{i}}(q_{i}X_{i}q_{i+1})$$ to $$^{k_{i}}Dr_{\tau _{i}}^{l_{i}}(q_{i}$$$$X_{i}q_{i+1})$$, where $$q_{m+1}=q,\ \forall i=1,\ldots ,m$$,

$$f\mid _{\bigcup \nolimits _{i=1}^{m}\ ^{k_{i}}Path_{\tau _{i}}^{l_{i}}(q_{i}X_{i}q_{i+1})}$$ is a bijective mapping from $$\bigcup \nolimits _{i=1}^{m}\ ^{k_{i}}Path_{\tau _{i}}^{l_{i}}(q_{i}X_{i}q_{i+1})$$ to $$\bigcup \nolimits _{i=1}^{m}\ ^{k_{i}}Dr_{\tau _{i}}^{l_{i}}$$$$(q_{i}X_{i}q_{i+1})$$. And so $$f\mid _{^{k+1}Path_{\omega }^{l}(pZq)}$$ is also bijective from $$^{k+1}Path_{\omega }^{l}(pZq)$$ to $$^{k+1}Dr_{\omega }^{l}(pZq)$$. Then the mapping *f* is bijective by mathematical induction from $$Path_{\omega }^{l}(pZq)$$ to $$Dr_{\omega }^{l}(pZq)$$.

Therefore $$|Path_{\omega }^{l}(pZq)|=|Dr_{\omega }^{l}(pZq)|$$.

Now we write *nl* as a shorthand for $l+\cdots +l$ (*n* summands) for every $l\in A$ and a positive integer *n*. Let $0l=0$. Then for every $\omega \in \Sigma ^{*}$, there always have $\langle rec(\mathcal {M})\rangle _{r}(\omega )=\sum \{\sigma (p)\cdot \vdash _{\mathcal {M}} ((p,\omega ,Z_{0}),(q,\varepsilon ,\varepsilon ))|p,q\in Q\}+ \sum \{\sigma (p)\cdot \vdash _{\mathcal {M}}^{t(\omega )}((p,\omega ,Z_{0}), (q,\varepsilon ,\varepsilon ))|p,q\in Q,t(\omega )\in Path_{\omega }^{l}(p,Z_{0},q),l\in A{\setminus }\{0\}\} =\sum \{I(p)\cdot \rho (p\Rightarrow [pZ_{0}q]\Rightarrow ^{L}\omega )|p,q\in Q\}+\sum \{|Path_{\omega }^{l}(pZ_{0}q)|(\sigma (p)\cdot l)|p,q\in Q,l\in A{\setminus }\{0\}\}=\sum \{I(p)\cdot \rho (p\Rightarrow [pZ_{0}q]\Rightarrow ^{L}\omega )|p,q\in Q\}+\sum \{|Dr_{\omega }^{l}(pZ_{0}q)|(I(p)\cdot l)|p,q\in Q,l\in A{\setminus }\{0\}\} =\sum \{I(p)\cdot \rho (p\Rightarrow [pZ_{0}q]\Rightarrow ^{L}\omega )|p,q\in Q\}+\sum \{I(p)\cdot \rho ([pZ_{0}q]\Rightarrow _{c(\omega )}^{*L}\omega )|p,q\in Q,c(\omega )\in Dr_{\omega }^{l}(pZ_{0}q), l\in A{\setminus }\{0\}\}=\langle G\rangle _{r}^{L}(\omega )$, and $\langle rec(\mathcal {M})\rangle _{b}(\omega )=\sum \{\sigma (p) \cdot (\sum \{\vdash _{\mathcal {M}}((p,\omega ,Z_{0}),(q,\varepsilon ,\varepsilon ))| q\in Q\})|p\in Q\}+ \sum \{\sigma (p)\cdot (\sum \{\vdash _{\mathcal {M}}^{t(\omega )} ((p,\omega ,Z_{0}),(q,\varepsilon ,\varepsilon ))| q\in Q,t(\omega )\in Path_{\omega }^{l}(p,Z_{0},q),l\in A{\setminus }\{0\}\})|p\in Q\} =\sum \{I(p)\cdot (\sum \{\rho (p\Rightarrow [pZ_{0}q]\Rightarrow ^{L}\omega )|q\in Q\})|p\in Q\}+\sum \{I(p)\cdot (\sum \{\rho ([pZ_{0}q]\Rightarrow _{c(\omega )}^{*L}\omega )|q\in Q,c(\omega )\in Dr_{\omega }^{l}(pZ_{0}q), l\in A{\setminus }\{0\}\})|p\in Q\}=\langle G\rangle _{b}^{L}(\omega )$.

Finally we will give two examples to show that in the basis of run semantics and breadth-first algebraic semantics, how to construct the equivalent machines for weighted context-free grammars and weighted pushdown automata over strong bimonoids respectively. In the first example we construct a weighted pushdown automaton over the underlying strong bimonoid equivalent to the given weighted context-free grammar over a strong bimonoid. In the second example we consider an equivalent weighted context-free grammar over the strong bimonoid when given a WPDA$^{\emptyset }$ over a strong bimonoid.

### *Example 1*

Let $G=(\{X,B\},\{a,b\},P,I)$ be a WCFG over a complete strong bimonoid *A*, where

$$P=\{X\overset{t_{1}}{\rightarrow }baXXa, X\overset{t_{2}}{\rightarrow }ab, B\overset{t_{3}}{\rightarrow }bBB, B\overset{t_{3}}{\rightarrow }ba\}$$, $$I(X)=t_{0}=I(B)$$, $$t_{0},t_{1},t_{2},t_{3}\in A{\setminus }\{0\}$$.

Next we construct a WPDA$$^{\emptyset }$$$$\mathcal {M}=(Q,\Sigma ,\Gamma ,\delta ,\sigma ,Z_{0},\emptyset )$$ over the strong bimonoid *A* as follows:

1. $$Q=\{X,B,q\}$$;
2. $$\Sigma =\{a,b\}$$;
3. $$\Gamma =\{a,b,X,B,Z_{0}\}$$;
4. $$\sigma (X)=\sigma (B)=t_{0}$$, $$\sigma (q)=0$$;
5. $$\delta (S,\varepsilon ,Z_{0},q,S)=1, \forall S\in \{X,B\}$$;
6. $$\delta (q,a,a,q,\varepsilon )=\delta (q,b,b,q,\varepsilon )=1$$;
7. $$\delta (q,\varepsilon ,X,q,baXXa)=t_{1}$$, $$\delta (q,\varepsilon ,X,q,ab)=t_{2}$$, $$\delta (q,\varepsilon ,B,q,bBB)=t_{3}$$, $$\delta (q,\varepsilon ,B,q,ba)=t_{3}$$.

Otherwise, $$\delta (p_{1},u,\gamma ,p_{2},\gamma _{1})=0$$.

Then by Proposition 8, we has $\langle rec(\mathcal {M})\rangle _{x}=\langle G\rangle _{x}^{L}, \forall x\in \{r,b\}$.

### *Example 2*

Let $\mathcal {M}=(Q,\Sigma ,\Gamma ,\delta ,\sigma ,Z_{0},\emptyset )$ be a WPDA$^{\emptyset }$ over a complete strong bimonoid *A*, where $Q=\{p,q,q_{1},q_{3}, q_{4},q_{5}, q_{6}\}$, $\Sigma =\{a,b\}$, $\Gamma =\{Z_{0},X,X_{1},X_{2},X_{3}\}$, $\sigma (p)=1$, $\sigma (y)=0,\forall y\in Q{\setminus }\{p\}$; suppose $t,t_{1},t_{2},t_{3},t_{4},t_{5},t_{6},t_{7}\in A{\setminus }\{0\}$, a map $\delta : Q\times (\Sigma \cup \{\varepsilon \})\times \Gamma \times Q\times \Gamma ^{*}\rightarrow A$ is given by, $\delta (p,a,Z_{0},q_{1},XX_{1})=t$, $\delta (q_{1},b,X,q,X_{2}X_{3})=t$, $\delta ( q,b,X_{2},q_{3},X_{2})=t_{1}$, $\delta (q,b,X_{2},q_{4},\varepsilon )=t_{2}$, $\delta (q_{3},a,X_{2},q_{3},\varepsilon )=t_{3}$, $\delta (q_{4},a,X_{3},q_{4},X_{3})=t_{4}$, $\delta (q_{3},a,X_{3},q_{5},\varepsilon )=t_{5}$, $\delta (q_{4},a,X_{3},q_{5},\varepsilon )=t_{6}$, $\delta (q_{5},a,X_{1},q_{6},\varepsilon )=t_{7}$, otherwise, $\delta (p_{1},u,\gamma ,p_{2},\gamma _{1})=0$.

By Proposition 9, construct a WCFG $G=(N,T,P,I)$ over *A* as follows:

$N=Q\cup \{[p_{1}Zp_{2}]|p_{1},p_{2}\in Q,Z\in \Gamma \}$, $T=\Sigma$, $I(p)=\sigma (p)$, $I(x)=0,\forall x\in N{\setminus }\{p\}$. *P* consists of the following productions:

1. $$p_{1}\overset{1}{\rightarrow }[p_{1}Z_{0}p_{2}],\forall p_{1},p_{2}\in Q$$;
2. $$[pZ_{0}p_{2}]\overset{t}{\rightarrow }a[q_{1}Xp_{1}][p_{1}X_{1}p_{2}]$$, $$[q_{1}Xp_{2}]\overset{t}{\rightarrow }b[qX_{2}p_{1}][p_{1}X_{3}p_{2}]$$, $$[qX_{2}p_{2}]\overset{t_{1}}{\rightarrow }b[q_{3}X_{2}p_{2}]$$, $$[q_{4}X_{3}p_{2}]\overset{t_{4}}{\rightarrow }a[q_{4}X_{3}p_{2}], \forall p_{1},p_{2}\in Q$$;
3. $$[qX_{2}q_{4}]\overset{t_{2}}{\rightarrow }b$$, $$[q_{3}X_{2}q_{3}]\overset{t_{3}}{\rightarrow }a$$, $$[q_{3}X_{3}q_{5}]\overset{t_{5}}{\rightarrow }a$$, $$[q_{4}X_{3}q_{5}]\overset{t_{6}}{\rightarrow }a$$, $$[q_{5}X_{1}q_{6}]\overset{t_{7}}{\rightarrow }a$$.

It follows by Proposition 9 that $\langle rec(\mathcal {M})\rangle _{x}=\langle G\rangle _{x}^{L},\ \forall x\in \{r,b\}$.

## Conclusions

In this paper, we considered firstly weighted pushdown automata and context-free grammars over strong bimonoids on the basis of run semantics and breadth-first algebraic semantics. The algebraic properties characterizations of these machines are investigated. Based on each semantics, weighted pushdown automata with empty stack and weighted context-free grammars over strong bimonoids are shown to be equivalent. Finally, two examples are presented to illustrate the proposed methods in this paper. These results obtained in this paper, which generalize the previous conclusions in literature (Qiu 2007b; Xing and Qiu 2009) to a certain extent, will lay the foundations for more detailed analysis of the applications such as in multi-valued model checking. Further studies will explore the normal context-free grammars over strong bimonoids based on some semantic ways. It would be interesting to see how much differently these context-free grammars’ behaviors. Also, it would be interesting to utilize weighted context-free grammars and weighted pushdown automata based on complete strong bimonoids to deal with modelling and control problems of fuzzy discrete event systems.
